# A Review of Heavy Metals in Coastal Surface Sediments from the Red Sea: Health-Ecological Risk Assessments

**DOI:** 10.3390/ijerph18062798

**Published:** 2021-03-10

**Authors:** Khalid Awadh Al-Mutairi, Chee Kong Yap

**Affiliations:** 1 Department of Biology, Faculty of Science, University of Tabuk, P.O. Box 741, Tabuk 47512, Saudi Arabia; kmutairi@ut.edu.sa; 2 Department of Biology, Faculty of Science, Universiti Putra Malaysia, UPM, Serdang 43400, Selangor, Malaysia

**Keywords:** Red Sea, heavy metals, sediments, ecological risks, human health risks

## Abstract

The heavy metal (HM) pollution in sediment is of serious concern, particularly in the Red Sea environment. This study aimed to review and compile data on the concentrations of four HMs (Cd, Cu, Pb, and Zn) in the coastal surface sediments from the Red Sea, mainly from Saudi Arabia, Egypt, and Yemen, published in the literature from 1992 to 2021. The coastal sediments included those from mangrove, estuaries, and intertidal ecosystems. It was found that the mean values of Cd, Cu, Pb, and Zn in coastal Red Sea sediments were elevated and localized in high human activity sites in comparison to the earth upper continental crust and to reference values for marine sediments. From the potential ecological risk index (PERI) aspect, 32 reports (47.1%) were categorized as ‘considerable ecological risk’ and 23 reports (33.8%) as ‘very high ecological risk’. From the human health risk assessment (HHRA) aspect, the non-carcinogenic risk (NCR) values (HI values < 1.0) of Cd, Cu, Pb, and Zn represented no NCR for the ingestion and the dermal contact routes for sediments from the Red Sea countries. The reassessment of the HM data cited in the literature allowed integrative and accurate comparisons of the PERI and HHRA data, which would be useful in the management and sustainable development of the Red Sea area, besides being a helpful database for future use. This warrants extensive and continuous monitoring studies to understand the current and the projected HM pollution situation and to propose possible protective and conservative measures in the future for the resource-rich Red Sea ecosystem.

## 1. Introduction

Monitoring studies of heavy metal (HM) pollution by using sediments are expected to increase in the future based on the increasing number of papers in the literature. Judging from the recent paper by Dash et al. [1] who presented the assessment of sediment pollution loadings and their ecological risks, such monitoring studies would be more pronounced and significant from the ecotoxicological and public health points of view. Information on HM pollution in sediment in the Red Sea ecosystem is important in order to understand the current situation and to propose possible remedial measures to overcome the increasing environmental stresses [2].

From 1964 until January 2021 based on a Scopus database search in January 2021, many studies (>4400 papers) in the literature reported on the HMs in the sediments from different countries (>125 countries with China topping the number with >1400 papers). From the monitoring and management points of view, this is an encouraging number and a positive trend because the number of such papers are increasing every year. However, from an effective comparison purpose, we can only confidently compare similar metals reported from different locations. 

The four metals namely Cd, Cu, Pb, and Zn were selected for review in this paper because of three reasons. Firstly, inconsistency of specific HM in the sediments reported in the literature. The only paper found based on Cd, Pb, Cu, and Zn was that reported by El-Sadaawy et al. [3] on sediments collected from the north-western part of the Egyptian coast of the Red Sea around Hurghada. They reported the PERI values (62.9–986) based on Cd, Pb, Cu, and Zn but not all the papers in the literature reported such values for these metals. Therefore, the comparison of the potential ecological risk index (PERI) based on a combination of other metals would be difficult. One study’s PERI value based on 5 metals would not be comparable to another based on 4, 6, or more metals. For example, Cheng and Yap [4] reported the PERI values (57.74–141) of mangrove surface sediments sampled from nine geographical sites in Peninsular Malaysia based on a combination of seven metals (As, Cd, Cu, Cr, Hg, Pb, and Zn), which were found as ‘low ecological risk’, Based on mangrove sediments from the Yanbu coast, Alharbi et al. [5] reported the PERI values (ranges of 8.22–85.1) based on 4 HMs (Cr, Cu, Ni, Pb, and Zn). Therefore, the PERI values of the above two studies are not comparable. 

Secondly, a lot of studies on the human health risk assessment (HHRA) of Cd, Cu, Pb, and Zn were reported in the literature. These four HMs consist of two essential (Cu and Zn) and two non-essential (Cd and Pb) metals, which can represent the whole group of HMs as seen in the Periodic Table. Kumar et al. [6] reviewed the HM data including Cd, Cu, Pb, and Zn in the sediments published from India and made HHRA of HMs based on the reviewed data. Wang et al. [7] assessed the non-carcinogenic risks (NCR) for nine HMs including Cd, Cu, Pb, and Zn, in the different sediment textures (Mud, Mud-Sand, and Sand) collected from seven estuarine wetlands across four provinces along the coastline of China. The above recent studies highlighted the human health risk (HHR) exposure to HMs even in the estuarine sediments and the significance of the NCR of Cd, Cu, Pb, and Zn to human beings associated with HMs in the surface sediment.

Thirdly, many studies conducted in Saudi Arabia [5], Yemen [8], and Egypt [9] did not report PERI values. In the case of research conducted in Haqal coastal waters where PERI was reported but not for HHRA [10].

All the literature above also never assessed the HHRA of Cd, Cu, Pb, and Zn in the surface sediments. However, the HMs in sediments may also re-enter the water and cause secondary contamination, leading to possible environmental effects and threats to human health [11]. In order to compare the PERI and HHRA values reported from different locations and different authors, the four commonly reported HMs were selected and reviewed in this paper.

### 1.1. Why Use the Potential Ecological Risk Index?

Firstly, the use of the potential ecological risk index (PERI), which was firstly proposed by Hakanson [12], had already received a total of 3682 citations based on a Scopus data (www.scopus.com) searched on 9 January 2021. The first and the latest citations were Hakanson [12], and Saleh and Marie [8], respectively. All the citations used a similar toxic response factor (Tr) for Cd, Cu, Pb, and Zn. This could be attributed to the fact that there were no recent studies available in the literature. However, the background levels used in the cited data for this paper were different. The background levels employed in the literature included the pre-industrial reference levels by Hakanson [13] and the upper continental crust (UCC) levels of the HMs proposed by Turekian and Wedepohl [14], and Wedepohl [15], Taylor and McLennan [16], and Rudnick and Gao [17]. In this paper, the background levels by Wedepohl [15] were used for the determination of PERI because it received extensive citations of 3555 so far in Scopus (until 2 March 2021).

Secondly, due to the inconsistency and comprehensiveness of risk assessments for HMs in surface sediments [18], the present study used the reported data of Cd, Cu, Pb, and Zn from the literature and re-analysed the potential ecological risk index (PERI) to make accurate comparative interpretations. This was because of the use of similar levels of background upper continental earth crust (UCC) by Wedepohl [15] of the four HMs besides the toxic response levels by Hakanson [12]. Furthermore, Yu et al. [18] reported that the assessment indices such as geoaccumulation index, and risk assessment code, were not consistent with one another in terms of predicting environmental risks caused by HMs in the sediments.

### 1.2. Why Focus on the Red Sea?

The Red Sea is unique among the world’s seas because there are no permanent streams flowing into it, and because only waves, often from the northwest, and occasional rain torrents add materials to its floor [19]. It is assumed that the Red Sea is a relatively unpolluted sea. However, it has been subjected to at least three sources of emissions: (i) petroleum emissions from tanker activities, oil fields, and refineries [10,20]; (ii) eutrophication from waste, sewage and phosphate extraction, loading operations [21]; and (iii) heavy metal pollution as a result of anthropogenic industrial pollution and the mining of deep hot brine muds in the central Red Sea [10,22,23]. These deep pockets include Zn, Cu, Al, Pb, As, and Hg-rich brines. These contaminants, including those from richly built coral reefs, fishery resources and tourism industries, pose major threats to the biological ecosystems within the Red Sea [2].

The Red Sea, which hosts some of the most active and diverse coral reefs, is a special body of water. Due to the hot and arid climate surrounding it, human populations along the coasts of the Red Sea were initially scarce, but this is changing with improved desalination methods, affordable resources, and increased economic activities in the coastal areas. In addition to rising coastal growth pressures on the reefs, global drivers, mainly ocean acidification and seawater heating, are threatening the region’s coral reefs [24]. The Red Sea is a marine area in Egypt that is economically significant. Over the past decades, its coastal region has been exposed to the anthropogenic effects of urbanization and economic growth [3].

A unique arid tropical ecosystem with surface sediments, consisting mainly of indigenous calcareous sediments of biogenic origin, characterizes the coastal climate of the Red Sea. Via active wind transport, sometimes active wadis, and various human operations, the sea is loaded with varying amounts of silicyclastic sediments. This type of coastal system must be handled in a way that varies from those in humid environments in terms of environmental pollution [25]. The Red Sea catchment area consists of sedimentary, granitic, and metamorphic rocks that are weathered and eroded by wind and seasonal floods from the Red Sea (coastal) waters [26]. Owing to increased human activities in the Red Sea coastal areas, these natural conditions have been modified [24]. These operations are the most common on the coast of Jeddah City and include refilling, dredging, discharge of waste, fishing, and spills of oil. In recent years, several sites in Jeddah have been studied to research the anthropogenic effects and impacts of pollution on the environmental conditions of lagoons and shorelines. [5,26,27,28,29,30].

Hanna [2] reported that over the last 50 years (1934–1984), the concentrations of most of the HMs (Co, Ni, V, Pb, Cu, Cd, and Zn) have increased due to natural contamination from hot brine pools or because of man-made oil pollution, HM mining, discharge of domestic, industrial waste and phosphate mining, and transport along the Red Sea coastal areas. Hanna [2] reported that the total Zn concentrations (mg/kg) in Red Sea sediments (Gulf of Suez, El-Ghardada, Safaga, and Qoseir) collected in 1934 ranged from 6 to 70 while those of samples collected in 1984 ranged from 15 to 100; and the mean values were 24 for the 1934 samples, and 40 for the 1984 samples. The total Cu concentrations (mg/kg) ranged from 2 to 41 (mean: 17.6) in the Red Sea sediments of the 1934 samples while those of the 1984 samples ranged from 13–80 (mean: 33.0). These results evidently indicated that the Zn and Cu levels of the Red Sea sediments had doubled over the 50 years. The total Pb concentrations (mg/kg) of the Red Sea sediments of 1934 ranged from 0.80–19.0 (mean: 3.00) while those of the 1984 samples ranged from 8.00–41.5 (mean: 15.2). This indicated that the total Pb concentration increased five times over 50 years. The total Cd concentrations (mg/kg) of the Red Sea sediments of 1934 ranged from 0.10–0.60 (mean: 0.40) while those of the 1984 samples ranged from 0.50–2.00 (mean: 1.10). This indicated that the total Cd concentration increased about 2–3 times in 50 years. 

Ahdy and Youssef [9] found that inadequate leisure facilities have resulted in numerous impacts on the coastal ecosystems facing some of the Red Sea’s north-western recreation projects. Industrial and human activities in the coastal region of Saudi Arabia have increased dramatically over the last three decades, resulting in the continuous invasion of various forms of contaminants, including HMs [31].

### 1.3. Objectives of the Study

For this paper, we compiled the data of Cd, Cu, Pb, and Zn in coastal sediments of the Red Sea from 1992 to 2021. We reviewed the literature and analyzed the data published by various authors to assess the HM status of the sediments of the coastal areas of the Red Sea published mainly from Saudi Arabia, Egypt, and Yemen. Therefore, the objectives of this paper were to review the HM concentrations in the coastal surface sediments from the Red Sea and to determine the PERI and human health risk assessment (HHRA) based on the reviewed data for effective comparisons and accurate interpretations. This was because the calculations of PERI and HHRA of the four HMs were based on similar exposure factors and reference values.

## 2. Methodology

### Data Collection

**Figure 1:**
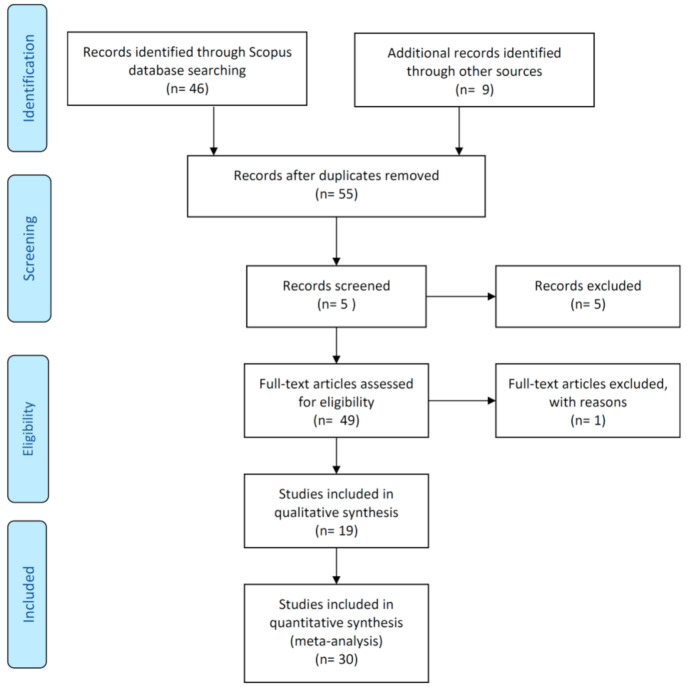
Flowchart of the Preferred Reporting Items for Systematic Reviews and Meta-Analyses (PRISMA) (adapted from Moher et al. [32]) used in the present study.

The systematic literature review (SLR) approach of the Preferred Reporting Items for Systematic Reviews and Meta-Analyses (PRISMA) by Moher et al. [32] was used in the current review research to add to the established body of information on HMs in the Red Sea sediments. PRISMA is an evidence-based norm of reporting that is useful for critical assessment. Overall, Figure 1 demonstrates the measures of the formal approach that have been modified for this review paper.

On 1 January 2021, a survey of the literature based on the Scopus database was conducted to arrive at a total of 46 published papers (excluded one comment paper; one reply to comment) where the keywords ‘Metals’, ‘Sediments’, and ‘Red Sea’ must be found in the title of the papers under the Scopus database. The database Scopus was used for literature analysis because it is the most common database for conducting literature searches. As on 29 April 2020, Scopus is the biggest dynamic reference information base explored for writings that incorporate logical diaries, books and gathering procedures [33]. These papers evaluated the scholastic distributions on the topics of ‘Metals’, ‘Sediments’, and ‘Red Sea’ that were accessible on the Scopus bibliographic information base. However, out of this 46, only 22 papers were selected for their data of Cd, Cu, Pb, and Zn because of the presentation and inclusion of the four HMs besides being relevant and representative of the study area in the Red Sea. 

In this paper, for the calculations of PERI and HHRA of Cd, Cu, Pb, and Zn, the 22 Scopus-indexed papers, and 8 non-Scopus papers were included because of relevancy. There were 15 papers from Egypt (12 Scopus and 3 non-Scopus papers; 36 reports), 11 papers from Saudi Arabia (7 Scopus and 4 non-Scopus papers; 22 reports), 3 papers from Yemen (2 Scopus and 1 non-Scopus papers; 9 reports), and 1 paper from Jordan (1 Scopus paper; 1 report) (Table 1). 

## 3. Data Treatment

### 3.1. Potential Ecological Risk Index

The potential ecological risk index (PERI) was used to determine the potential risk of the HMs in the sediments to the ecology. This PERI was proposed by Hakanson [12]. The tabulations of PERI were done in the series of formulas. 

Firstly, the calculation of contamination factor (Cf) was based on the pollution of a single metal factor in Equation (1):

(1) $$\mathrm{Cf}=\frac{C_{s}}{C_{B}}$$

where C_s_ is the concentration of HM in sediments. C_B_ is the background value of each HM in the sediments. The background levels are the background concentrations in the earth’s upper continental crust (UCC), which are Cd (0.10 mg/kg), Cu (14.3 mg/kg), Pb (17.0 mg/kg), and Zn (52.0 mg/kg) based on Wedepohl [15].

Secondly, the calculation of ecological risk (Er), which is the potential ecological risk of a single element, was based on Equation (2):

(2) $$\mathrm{Er}=T_{R}\times \mathrm{Cf}$$

where T_R_ is the toxic response factor of a single metal. The T_R_ values used in the present study are Cd = 30.0, Cu = 5.00, Pb = 5.00, and Zn = 1.00, according to Hakanson [12]. According to Hakanson [12], five classifications for the Er are ‘low potential ecological risk’ (Er < 40); ‘moderate potential ecological risk’ (40 ≤ Er < 80); ‘considerable potential ecological risk’(80 ≤ Er < 160); ‘high potential ecological risk’ (160 ≤ Er < 320), and ‘very high ecological risk’ (Er ≥ 320).

Lastly, the summation of all the Er values for each metal would result in the PERI value, which was calculated based on Equation (3):

(3) PERI=∑Er

According to Hakanson [12], four classifications for PERI values are ‘low ecological risk’ (PERI < 150); ‘moderate ecological risk’(150 ≤ PERI < 300); ‘considerable ecological risk’ (300 ≤ PERI < 600), and ‘very high ecological risk’ (PERI ≥ 600).

### 3.2. Human Health Risk Assessment 

In general, humans have been exposed to HM sediments through three main routes: ingestion, inhalation, and dermal contact [57]. However, at the land-sea boundary, where the marine sediments were located and shared the coastal waters, the dosage was not determined by sediment inhalation. The risk assessment was performed in this study by calculating the sediment-related index based on two key pathways: ingestion and dermal contact [7,58].

Human health risk assessment (HHRA) of sediments is generally utilized to measure non-carcinogenic risk (NCR) to humans by means of three exposure pathways namely ingestion, inhalation, and dermal contact. The methodology utilized for the HHRA depended on the guidelines and Exposure Factors Handbook of the US Environmental Protection Agency [57,59,60,61]. The average daily doses (ADDs) (mg/kg day) of potentially toxic metals through the ingestion (ADD_ing_), inhalation (ADD_inh_), and dermal contact (ADD_der_) for both children and adults were calculated by using Equations (4) and (5) as shown below:

(4) $${\mathrm{ADD}}_{\mathrm{ing}}=C_{\mathrm{sediment}}\left(\frac{\mathrm{IngR}\;\times \mathrm{EF}\;\times \mathrm{ED}}{\mathrm{BW}\;\times \;\mathrm{AT}}\right)\;\times {-6}^{10}$$

(5) $${\mathrm{ADD}}_{\mathrm{der}}=C_{\mathrm{sediment}}\left(\frac{\mathrm{SA}\;\times \mathrm{AF}\;\times \;\mathrm{ABS}\;\times \mathrm{EF}\;\times \mathrm{ED}}{\mathrm{BW}\;\times \;\mathrm{AT}}\right)\;\times {-6}^{10}$$

where ADD_ing_, and ADD_der_ are the daily amounts of exposure to metals (mg/kg day) through ingestion, and dermal contact, respectively. In this study, NCR of HMs were assessed by using the hazard quotient (HQ) and hazard index (HI) [62]. Definition, exposure factors, and reference values used to estimate the intake values and health risks of HMs in sediments are presented in Table 2. 

The HQ is the proportion of the ADD of a metal to its reference dose (RfD) for similar exposure pathway(s) [59]. The RfD (mg/kg day) is the maximum daily dose of a metal from a particular exposure pathway, for both children and adults, that is accepted not to prompt a considerable risk of harmful effects to sensitive individuals during a life time. For Cd, the RfD (mg/kg day) values used in the present study were 1.00 × 10^−3^ and 1.00 × 10^−5^ for ingestion, and dermal contact, respectively. For Cu, the RfD (mg/kg day) values used in the present study were 4.00 × 10^−2^ and 1.20 × 10^−2^ for ingestion, and dermal contact, respectively. For Pb, the RfD (mg/kg day) values used in the present study were 3.50 × 10^−3^ and 5.25 × 10^−4^ for ingestion, and dermal contact, respectively. For Zn, the RfD (mg/kg day) values used in the present study were 3.00 × 10^−1^ and 6.00 × 10^−2^ for ingestion, and dermal contact, respectively [66]. If the ADD was less than the RfD value (HQ ≤ 1), it was viewed as that there would be no adverse health effects, while if the ADD surpassed the RfD value (HQ > 1), it was likely that there would be harmful health effects [59,61]. 

The NCR is assessed by HI, which is the summation of the HQs in the two exposure pathways [67,68,69]. A HI of <1.0 was expected to show that there was no significant risk of non-carcinogenic effects. A HI of >1.0 was expected to show that there was a possible occurrence of non-carcinogenic effects. The probability of non-carcinogenic effects has positive connection with the increment of HI value [66]. The HI is calculated as Equation (6):

(6) $$\mathrm{HI}=\sum {\mathrm{HQ}}_{i}=\;\sum \left(\frac{{\mathrm{ADD}}_{i}}{{\mathrm{RfD}}_{i}}\right)\;$$

### 3.3. Data Analysis

All statistical calculations were done by using the KaleidaGraph (Version 3.08, Sygnergy Software, Eden Prairie, MN, USA).

## 4. Results and Discussion

### 4.1. Heavy Metals in Sediments

The concentrations of HMs in the sediments reported from the Red Sea countries are presented in Table 1. Based on 36 reports from Red Sea Egypt, the metal concentrations (mg/kg dry weight) ranges were 0.02–21.6 for Cd (mean: 2.18), 0.05–454 for Cu (mean: 30.8), 0.01–865 for Pb (mean: 57.8), and 0.01–515 for Zn (mean: 77.2). Based on 22 reports from Red Sea Saudi Arabia, the metal concentrations (mg/kg dry weight) ranges were 0.02–3.95 for Cd (mean: 1.04), 0.17–111 for Cu (mean: 29.8), 0.05–240 for Pb (mean: 39.7), and 3.82–532 for Zn (mean: 74.5). Based on nine reports from Red Sea Yemen, the metal concentrations (mg/kg dry weight) ranges were 0.20–7.30 for Cd (mean: 1.77), 3.60–84.8 for Cu (mean: 28.4), 2.40–17.7 for Pb (mean: 5.49), and 1.60–91.7 for Zn (mean: 19.8). The only report from Jordan documented concentrations of 8.00, 42.4, 96.7 and 3.33 for Cu, Zn, Pb, and Cd, respectively. 

**Figure 2:**
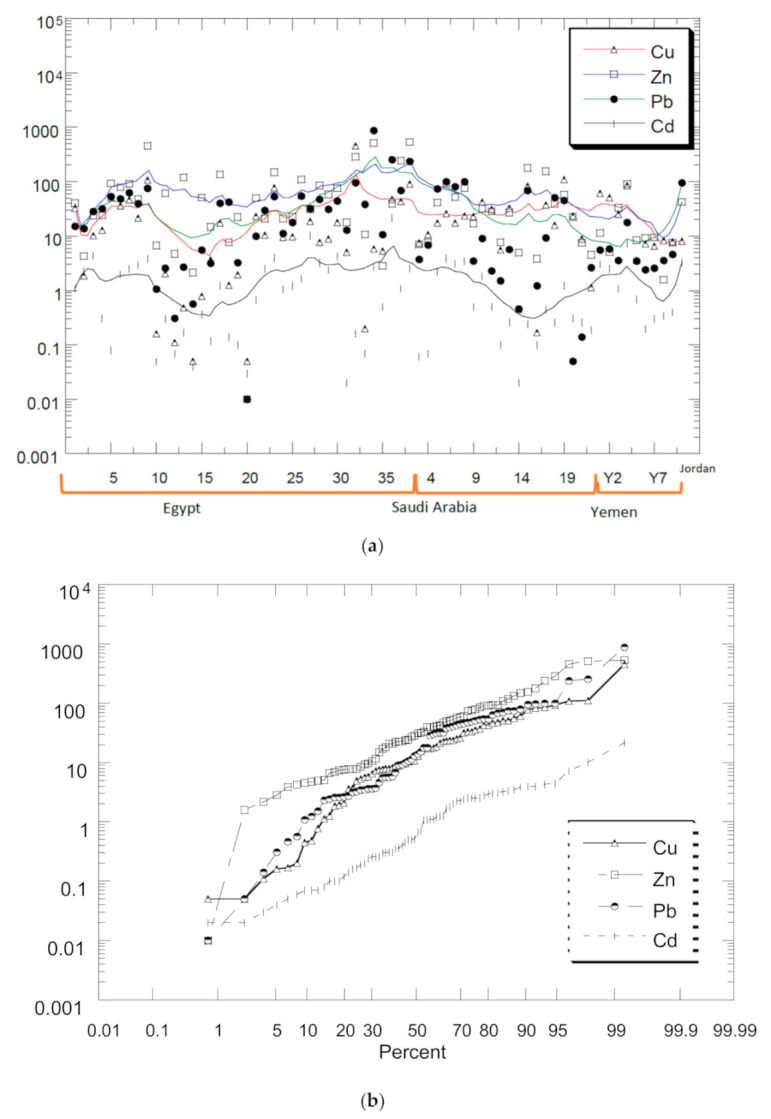
Comparisons of the distribution levels of Cd, Cu, Pb, and Zn reported from (**a**) the four countries in the Red Sea and (**b**) their probabilities of percentages. The log_10_ Y-axis represents the metal concentrations (mg/kg dry weight) for both (**a**) and (**b**).

The descriptive statistics of HM concentrations (mg/kg dry weight) in the surface sediments of different countries in the Red Sea, compared to establish background values, are presented in Table 3. The comparisons of the distribution levels of Cd, Cu, Pb, and Zn reported from the Red Sea (Figure 2a). Their probabilities of percentages in Figure 2b indicate the overall metal distribution followed: Zn > Pb > Cu > Cd.

Based on Table 3, similar metal distributions were found for reports from Egypt and Saudi Arabia. However, Yemen followed the order: Cu > Zn > Pb > Cd while Jordan followed Pb > Zn > Cu > Cd. The natural background levels of UCC by Wedepohl [15] for the metal distributions followed: Zn > Cu > Pb > Cd. Therefore, none of the above metal distributions followed the UCC by Wedepohl [15]. This indicated metal redistributions of the natural abundances of Cd, Cu, Pb, and Zn. This could be attributed to anthropogenic inputs of elevated Pb levels in Egypt (mean: 57.8; 3.85 times higher than Pb UCC level) and Saudi Arabia (mean: 39.7; 2.65 times higher than Pb UCC level), and elevated levels of Pb (6.44 times higher than Pb UCC level) and Cd (33.3 times higher than Cd UCC level) in Jordan.

When compared to the reference values (Table 3), the overall mean Cd of the Red Sea countries were all above those of the six reference values. Except for Wedepohl [70], the overall mean Cu of the Red Sea countries were all below those of the pre-industrial reference Cu levels by Hakanson [12], UCC limits by Rudnick and Gao [17], Taylor and McLennan [16], Wedepohl [15], and the background average shale by Turekian and Wedepohl [14]. However, Cu reports from Jordan were also below those of all the six reference values. 

The overall mean Zn of the Red Sea countries were all below those of the pre-industrial Zn reference values by Hakanson [12] and Turekian and Wedepohl [14]. The mean Zn values from Egypt and Saudi Arabia were slightly higher than those of the UCC Zn limits by Rudnick and Gao [17], Taylor and McLennan [16], and Wedepohl [15,70]. The mean Zn values from Yemen and Jordan were all below those of all the six reference values.

Except for Yemen, the overall mean Pb of the Red Sea countries were all above those of all the six reference values. Most significantly, the elevated Pb level from Jordan was 1.38, 5.69, 5.69, 4.83, 5.69, and 6.44 times higher in comparison to Hakanson [12], Rudnick and Gao [17], Taylor and McLennan [16], Turekian and Wedepohl [14], Wedepohl [70], and Wedepohl [15], respectively.

### 4.2. Potential Ecological Risk Index

**Figure 3:**
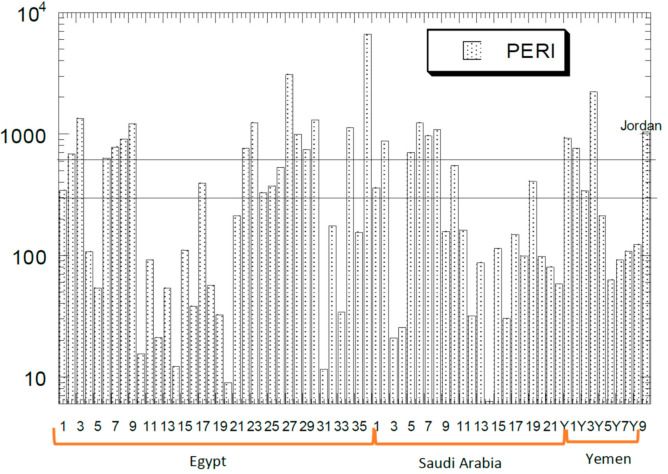
Comparisons of potential ecological risk index (PERI) values based on a combination of the ecological risks of Cd, Cu, Pb and Zn in the Red Sea countries. The lower and upper vertical lines indicate ‘considerable ecological risk’ (300 ≤ PERI < 600) and ‘very high ecological risk’ (PERI ≥ 600), respectively, according to Hakanson [12]. The log_10_ Y-axis represents the ecological risk values of PERI. X-axis represents the Red Sea countries investigated in this study.

Values of Cf and Er for Cd, Cu, Pb, and Zn, and PERI on sediments in the Red Sea countries are presented in Table S1. Comparisons of the PERI values based on a combination of the ecological risks of Cd, Cu, Pb, and Zn in the Red Sea countries are presented in Figure 3. Out of 68 reports, there were 32 reports (47.1%) exceeding PERI > 300 with ‘considerable ecological risk’ while there were 23 reports (33.8%) exceeding PERI > 600 with ‘very high ecological risk’, according to Hakanson [12]. 

The overall mean values of PERI based on all the 68 sites were Cd (583), Cu (43.3), Ni (1.42), Pb (38.4), and Zn (7.88) (Figure 3). Therefore, the PERI for single metals demonstrated that the severity of pollution of the six metals diminished in the following succession: Cd > Cu > Pb > Zn. The present finding was comparable to the sequence based on Anshan soils, which was Cd > Cu > Pb > Ni > Zn, as reported by Qing et al. [62]. 

In this study, Cd in the sediments in the Red Sea countries had a high contribution to the increment of the PERI. The percentage of the Cd Er contribution of PERI was in the range of 27.4–99.9% (mean: 91.6%). Hence, the reason why the majority of the sampling sites were having ‘moderate’ to ‘very high ecological risk’ was mainly due to Cd. The contributions of Er to PERI from the other HMs were much lower than that of Cd, namely Cu (0.10–51.8%; mean = 2.85%), Pb (0.00–37.4%; mean = 5.02%), and Zn (0.00–3.40%; mean = 0.49%). This agreed with the report by Qing et al. [62] which was 90% Cd Er. These outcomes showed the ‘high potential ecological risk’ that Cd could pose to the human body and to the biological ecosystem.

### 4.3. Human Health Risk Assessment

The HHRA results due to HM exposures of sediments from the Red Sea cited from Egypt, Saudi Arabia, Yemen, and Jordan are presented in Tables S2–S5. The overall statistics of the values of hazard quotient dermal (HQ_dermal_) and hazard quotient ingestion (HQ_ing_), and hazard index of Cd, Cu, Pb, and Zn for children and adults from the present study are presented in Table 4. 

#### 4.3.1. Cd

For children Cd (S2), based on the mean values of the four Red Sea countries, the HQ_ing_ values ranged from 2.62 × 10^−4^ to 3.79 × 10^−1^, and the HQ_dermal_ values ranged from 4.19 × 10^−5^ to 6.06 × 10^−2^. The HI values for children Cd ranged from 3.04 × 10^−4^ to 4.39 × 10^−1^. For adult Cd (S2), based on the mean values of the four Red Sea countries, the HQ_ing_ values ranged from 3.52 × 10^−5^ to 3.80 × 10^−2^, and the HQ_dermal_ values ranged from 1.07 × 10^−4^ to 1.16 × 10^−1^. The HI values for adult Cd ranged from 1.42 × 10^−4^ to 1.54 × 10^−1^. The Cd values of HQ_ing_ and HQ_der_ were higher in children than those in adults. The Cd HI values for both children and adults were lower than 1 in all the reports, indicating limited non-carcinogenic risk from Cd in Red Sea countries. 

As a transition element, Cd acts as a cumulative poison in the system. It is listed as one of the 129 priority contaminants by the EPA and is among the 25 hazardous substances listed. In addition, there is an international agreement not to pour Cd into the sea, as it is included in the Black List [71]. Via windblown transport of soil particles and volcanic emissions, the natural sources of Cd contribute 10–30 percent. By processing and using Cd, Cu, and Ni in smelting, and the atmospheric loading [72] most of which are deposited in the bottom sediments, the main source of Cd to the marine environment is predominantly anthropogenic [71]. Elevated levels of Cd were observed in the collected core sediments. This showed that this metal had an anthropogenic source in sediments that might involve the discharge of refining waste and untreated sewage effluents [73]. Badr et al. [31] stated that Cd’s vertical distribution pattern along the cores collected showed fluctuations, especially in cores I, II, III, and V. This indicated that Cd’s anthropogenic signal in these cores was relatively large. In addition, it is well known that Cd is prone to redox changes and it is known to be soluble under oxygenated conditions and to instantly precipitate where post-oxic conditions are encountered [74].

Ahdy and Youssef [9] stated that the results showed that Cd was the only metal in the north-western part of the Red Sea that posed a high risk to the environment, according to the Risk Assessment Code (RAC). Based on the Jeddah Red Sea coastal area surface sediments in Saudi Arabia, Al-Mur [26] found that the Cd risk assessment code showed a medium risk in five sediment samples from the northern and southern regions and a high risk in the other sediment samples for the aquatic climate.

Alzahrani et al. [75] stated that the maximum Cd concentration surpassed its threshold for toxic impact, revealing a harmful risk in the sediments to biota. The geo-accumulation index showed that mangrove sediments ranged from moderately to heavily contaminated with Cd in Al-Haridhah and moderately contaminated in South Jeddah, Rabigh, Duba, and the wastewater treatment plant near Jazan. The ERI revealed that the mangrove ecosystem might be posed a relatively very high risk due to Cd. This study highlighted the possibility of developing a coastal aquatic ecosystem management structure along Saudi Arabia’s Red Sea coast. Badr et al. [31] reported that Cd concentrations showed high variations in the depth of the patterns of Al, Fe, and Mn, suggesting land-based sources of this component for the areas studied. El-Said and Youssef [76] confirmed that the Cd risk affected the selected mangrove ecosystems collected from the Egyptian Red Sea shoreline.

#### 4.3.2. Cu 

For children Cu (S3), based on the mean values of the four Red Sea countries, the HQ_ing_ values ranged from 1.64 × 10^−5^ to 1.49 × 10^−1^, and the HQ_dermal_ values ranged from 8.74 × 10^−8^ to 7.94 × 10^−4^. The HI values for children Cu ranged from 1.65 × 10^−5^ to 1.50 × 10^−1^. For adult Cu (S3), based on the mean values of the four Red Sea countries, the HQ_ing_ values ranged from 2.20 × 10^−6^ to 2.00 × 10^−2^, and the HQ_dermal_ values ranged from 2.23 × 10^−7^ to 2.03 × 10^−3^. The HI values for adult Cu ranged from 2.42 × 10^−6^ to 2.20 × 10^−2^. The Cu values of HQ_ing_ and HQ_der_ were higher in children than in adults. The Cu HI values for both children and adults were lower than 1 in all the reports, indicating limited non-carcinogenic risk from Cu in Red Sea countries. 

Cu is one of the most common urban runoff-related pollutants. Sewage sludge dumpsites, industrial waste spill, and antifouling paints provided significant anthropogenic copper inputs into estuarine and coastal waters [72]. The copper concentration was found to cross 50 mg/kg in relatively clean sediment [77]. The EPA classifies sediments with higher concentrations than 60 mg/kg as polluted sediments [78]. Badr et al. [31] recorded the overall average Cu concentration of 21.3 mg/kg in the three areas analysed on the basis of the core sediments of certain Red Sea coastal areas. Hence, the sediments could be considered uncontaminated by Cu. In addition, the overall observed concentration of 28.3 mg/kg was well below the background concentration. Their results revealed the presence of recent anthropogenic inputs to this region and the addition of Cu from small sources to the marine sediments as antifouling paint from ships. 

#### 4.3.3. Pb 

For children Pb (S4), based on the mean values of the four Red Sea countries, the HQ_ing_ values ranged from 2.59 × 10^−5^ to 3.20, and the HQ_dermal_ values ranged from 2.80 × 10^−7^ to 3.46 × 10^−2^. The HI values for children Pb ranged from 2.62 × 10^−5^ to 3.24. For adult Pb (S4), based on the mean values of the four Red Sea countries, the HQ_ing_ values ranged from 3.48 × 10^−6^ to 4.30 × 10^−1^, and the HQ_dermal_ values ranged from 7.14 × 10^−7^ to 8.82 × 10^−2^. The HI values for children Pb ranged from 4.19 × 10^−6^ to 5.18 × 10^−1^. The Pb values of HQ_ing_ and HQ_dermal_ were higher in children than in adults. Except for report no. 34, the Pb HI values for both children and adults were lower than 1 in all the reports, indicating limited non-carcinogenic risk from Pb in Red Sea countries. Report no. 34 had the maximum Pb level (865 mg/kg) in the Hurghada region as reported by El-Sadaawy et al. [3].

All Pb compounds, especially tetraethyl lead, are potentially harmful or toxic [79]. It is classified as a carcinogenic material by the EPA. Badr et al. [31] reported that the concentrations of Pb in the analysed sediments showed high values in all cores, with minimum and maximum values in the range of 68.23 μg/g at the 15–20 cm core I to 109 μg/g at the 30–35 cm core III. Elevated Pb levels were attributed by Abu-Hilal [80] and Laxen [81] to several sources, such as boat exhaust systems, oil spillage, and other petroleum products from mechanized fishing vessels, and sewage effluent discharge into the water. All of these sources occurred in the areas studied. In addition to these sources, the atmospheric input of Pb produced by the emission of automotive exhaust emissions were important sources for areas near highways and city roads of the investigated areas. Frignani et al. [82] found that, by atmospheric inputs, Cd, Pb, and Zn were often added to the marine environment. Their research also found that both land-based activities and the use of leaded gasoline have increased over the last four decades.

Badr et al. [31] stated that in the bottom layers of the cores in Jeddah, elevated Pb concentrations were registered, suggesting the most drastic increases in gasoline use in the early 1970s. The following sequence of the measured CF values were found: Cd > Pb > Ni > Cu > Zn > Cr > Mn for all the studied areas.

#### 4.3.4. Zn

For children Zn (S5), based on the mean values of the four Red Sea countries, the HQ_ing_ values ranged from 4.37 × 10^−7^ to 2.32 × 10^−2^, and the HQ_dermal_ values ranged from 3.50 × 10^−9^ to 1.86 × 10^−4^. The HI values for children Zn ranged from 4.40 × 10^−7^ to 2.34 × 10^−2^. For adult Zn (S5), based on the mean values of the four Red Sea countries, the HQ_ing_ values ranged from 5.86 × 10^−8^ to 3.12 × 10^−3^, and the HQ_dermal_ values ranged from 8.93 × 10^−9^ to 4.75 × 10^−4^. The HI values for adult Zn ranged from 6.76 × 10^−8^ to 3.59 × 10^−3^. The Zn values of HQ_ing_ and HQ_dermal_ were higher in children than those in adults. The Zn HI values for both children and adults were lower than 1 in the all reports, indicating limited non-carcinogenic risk from Zn in the Red Sea countries. 

Zn is a naturally abundant element present in agricultural products, food waste, pesticides, as well as antifouling paints as a common contaminant. The down core profiles of Zn indicated variations in most cores of the region under investigation [31]. The same finding was recognized in sub-tidal sediments in NW Spain by Rubio et al. [83]. They reported the relationship of this result to Zn’s upward migration during the degradation of organic matter. Pattan et al. [84] found that in all sediment forms, including the coarse-grained ones, Cu, Ni, and Zn followed a comparable Mn pattern. In addition, Rubio et al. [83] proposed that during deposition, Zn was fixed primarily to the oxyhydroxides.

Dar [85] stated that the geo-accumulation factor of Zn was more significant compared to the other metals on the basis of sediments from the northern part of Safaga Bay. The bay was graded for Zn as being moderately to highly polluted, while with the other metals it was unpolluted to moderately polluted.

### 4.4. Exposure Behaviours of Heavy Metals

It was shown that the two different exposure pathways of Cd, Cu, Pb, and Zn for children and adults diminished in the following order: ingestion > dermal contact. The contributions of HQ_ing_ to HI (total risk of non-carcinogenic) were the highest for Zn (99.2% and 86.8% for children and adults, respectively), then Pb (98.9% and 83.0% for children and adults, respectively), Cu (99.5% and 90.8% for children and adults, respectively), and Cd (86.2% children). However, the Cd contribution of HQ_ing_ to HI was only 24.7% for adults. The highest Cd contribution (75.2%) to HI for adults was found in HQ_dermal_. These percentages were comparable to those reported in Anshan by Qing et al. [62], namely an average of 96.5% for children and 72.5% for adults based on Cr, Cd, Cu, Pb, Ni, and Zn. This emphatically showed that ingestion was the fundamental exposure pathway to undermine human health. 

By comparing the HI values for children and adults, it could be summarized that children had higher chances of NCR from HMs in the sediments of the Red Sea countries. According to the USEPA [57], if HI < 1, the exposed population would not show obvious adverse health effects [86]. The child group was generally exposed to greater risk of the adverse health effects from the influence of the contaminants. The results estimated that the child group risk was mostly caused by dermal absorption of the contaminants. The increment of the non-carcinogenic health risk was directly related to the exposed skin areas on the human body. Children generally have higher health risk exposure to the surrounding pollutants due to their behaviour and physiology. The higher NCR in children than adults is generally because of their pica behaviour and hand or finger sucking [87]. They have higher hand to mouth activities, higher respiration rates, and increased gastrointestinal absorption of some substances [88]. Higher health risks of HMs in children than in adults were reported in the literature [89,90]. 

### 4.5. Red Sea Coast of Jordan

The only paper reported in Jordanian Red Sea coast in this paper was that by Abu-Hilal and Badran [56]. They collected sediments in the northeastern portion of the Gulf of Aqaba and found elevated levels of Cd, Co, Cu, Fe, Mn, Pb, and Zn. These elevations were contributed by industrial discharges in the southern section of the study area, local discharges from accumulated phosphate rock particles, raw sewage, old barges, ship operations in the port area, and sewage outlets. They identified the sources of pollution as localized and also speculated that significant effects on marine life in the wider areas around the major sources of pollution in the northern portion of the Gulf of Aqaba in Jordan could be happening in future. 

### 4.6. Red Sea Coast of Sudan

Idris [91] investigated HM (Mn, Fe, Ni, Cu, Zn, and Pb) pollution in the sediments from Port-Sudan and Sawakin ports. Idris et al. [92] stated two sources (crustal and anthropogenic) in fine sediment grains in both harbours. Idris et al. [92] also reported that the fringing reefs on the southern side of Port-Sudan port had the highest order of magnitude of heavy metal pollution. This was mainly caused by discharges from oil refineries, industry, shipping activities, and domestic wastes. This was consistent with the findings of Sakai et al. [93], which, in addition to Cr and Cd, recorded emissions from oil refining activities by Fe, Ni, Cu, Zn, and Pb. Besides, man-induced industrial activities also contributed to the high heavy metal contents of sediments found in the northeastern part of the Port Sudan. 

### 4.7. Red Sea Coast of Yemen

Al-Shiwafi et al. [55] confirmed that the coastal ecosystems of Yemen’s Red Sea coast were still categorized as uncontaminated. Okbah et al. [94] concluded that in most countries, except in the eastern region, the fractionation of Pb had a medium risk, which led to a high risk to the Red Sea coast. Saleh and Marie [8] reported that Fe > Cu > Ni > Pb > Cd was the concentration order of the metals in the sediment of Hodeida coast. In addition, elevated levels of HMs were found in front of Al-Hodeidah City, was caused by the atmospheric contribution of local particulates from motor vehicles and from the mountain regions that drained their water through various valleys from the Yemen highlands to the Red Sea [53]. Therefore, the above studies concluded that the HM pollution in the Al-Hodeidah shore was still localized, especially near the sewage discharge sites and the port of Al-Hodeidah.

### 4.8. Red Sea Coast of Egypt

#### 4.8.1. Gulf of Suez and Gulf of Aqaba

Hamed and El-Moselhy [95] found that the northern part (Suez Bay) was polluted by HMs from Suez City, which received sewage and industrial effluents. However, the HM levels in Aqaba Gulf and the Red Sea proper were relatively low. Based on the western side of the northern part of the Gulf of Suez, El-Moselhy and Gabal [34] revealed that at stations affected by different sources of contamination, such as harbours, and sewage and industrial drains, the highest values of the studied metals were found. The lowest concentrations, by comparison, were found far away from any source of emissions. Masoud et al. [43] found that that the anthropogenic origin of Pb in the Gulf of Suez was due to human activities. 

El Nemr et al. [37] reported that Pb and Zn in the surface sediments collected from the Suez Gulf, Aqaba Gulf, and Middle Red Sea were mainly attributed to natural origins, while Cd was seriously affected, with moderate ecological risk, by human activities. Collection from beaches along the Ras-Gharib coast of the Gulf of Suez, Mostafa et al. [96] found that the degree of metal contamination was caused by anthropogenic activities (terrigenous sediments transported by some wadis in the General Beach region to the marine environment, oil spills resulting from exploration and production by the General Petroleum Company) and/or natural impacts. Based on surface sediments collected from the Suez Gulf, Aqaba Gulf, and Red Sea Proper, Salem et al. [40] reported that the studied HMs (Fe, Zn, Mn, Cu, Ni, Pb, Cd, Co, Cr, and Hg) did not pose any environmental risk to all of the stations investigated, with the exception of Marsa Alam and El-Quseir, which might have environmental risks due to Cr and Ni.

#### 4.8.2. Hurghada Area

Based on bottom sediments collected from Mabahiss Bay (north of the Hurghada Region), Attia and Ghrefat [45] concluded that the high content of Pb, Cd, and Co in the study area was the result of a number of anthropogenic activities, including dredging, land filling, localized oil contamination, the use of antifouling and anti-corrosive paints from fishing and tourist boats, and the discharge of waste from a variety of sources in that area. El-Sadaawy et al. [3] found that the Hurghada area was at very high-risk levels except for two stations at considerable risk.

Youssef et al. [97] showed that the Makadi Bay sediments (Hurghada) were essentially unpolluted by metals. Dar [85] revealed that Safaga Bay (located in the western side of the Red Sea 50 km south of Hurghada City) was categorized as low enrichment, indicative of no or minimal contamination, substantial enrichment, suggestive of a significant sequence of metal pollution signals: Zn > Cu > Pb.

In selected areas along the Hurghada coast, Mansour et al. [41] reported that the surface sediments showed high total concentrations of Pb and Zn in the transect of the Desert Rose Resort, Cd in the transect of El-Samaka Village and Abu-Shaar, and Cu in the transect of the Tourist Harbour. Comparing the current findings with other regional data and other global areas, it was clear that metal pollution was still localized and low in the Hurghada sector. 

Nour [30] discovered that the site of Hurghada was highly enriched with Pb, Cu, Zn, and Ni. In addition, the site of Quseir was highly enriched with Cd and with Pb. The site of Um al-Sid was intensely enriched with Cd and Pb. The Ras Mohamed site, meanwhile, was severely enriched with Pb and nearly uncontaminated with the other metals. Due to the weathering mechanism for the nearby beaches and mountains, ship repairs, industrial operations, waste water, and traffic exhaust, the HMs would reach the studied environment via terrigenous and anthropogenic sources.

#### 4.8.3. Wadi El-Gemal Coasts

Madkour et al. [44] showed that the high concentrations of HMs in the marine sediments were especially influenced by the high contribution of the Wadi El-Gemal stream of terrigenous materials. Their work showed how much natural inputs from this wadi impacted the marine sediments. El-Taher and Madkour [35] made no conclusion on the overall status of the HM emissions based on marine sediments collected from Wadi El-Hamra, Wadi El-Esh, Wadi Abu-Shaar, Wadi El-Gemal, and Wadi Khashir (Hamata). El-Taher et al. [36] later concluded that the allowable limits suggested by the Canadian Guidelines on environmental quality were exceeded by Fe, Mn, Ni, Co, Zn, Cu, Pb, and Cd.

#### 4.8.4. Shalateen Coasts

Nour et al. [38] concluded that the levels of Pb and Zn were largely due to anthropogenic sources, while Cu was attributed to a mixture of natural and anthropogenic sources. They revealed that human activities including fishing operations, antifouling paints, runoffs, desalination plants, industries, and dissolution of carbonate sediments were the potential sources of pollution. Dar et al. [98] found that higher levels of Cu and Pb were due to the continuous reworking processes in the surface sediments collected from Hamrawin Bay. 

### 4.9. Red Sea Coast of Saudi Arabia

#### 4.9.1. Coasts of Duba, Sharm, and Al-Wajh

Kahal et al. [47] reported that the average values for Cu and Zn in the sediments from the coastline between Duba and Sharma were higher than those reported for the Gulf of Aqaba and certain coasts of the Caspian Sea. The potential anthropogenic sources were landfilling, cement factory and the port of Duba, the Duba refinery station, and the tourist resort. Possible sources in the northern part were shipping and transport activities in the central area. Youssef et al. [27] reported a strong anthropogenic supply of Cd while Cu showed moderate anthropogenic inputs from urban and industrial activities and activities in the Wadi Haramel, Wadi Antar, Wadi Dumaygh, north of Al-Wajh. 

#### 4.9.2. Jeddah Coasts

Al-Mur et al. [99] reported that the Pollution Load Index was higher in the two locations closer to central Jeddah, where power and desalination plants and waste water release were known. Based on sediments collected from the coastal region of Jeddah, El Sayed and Basaham [100] reported the geochemical residual fraction Pb was very low and often entirely absent. This indicated that Pb was mainly contributed by Pb anthropogenic sources. El Zokm et al. [101] reported relatively high levels (mg/kg) of Cd (1.08–2.55), Cu (43.3–91.7), Pb (68.3–240), and Zn (241–532) based on the sediments of Jeddah Coast.

Ghandour et al. [25] recorded natural and anthropogenic controls at Sharm Obhur on the sediment composition of an arid coastal climate (about 35 km to the north of Jeddah City). They concluded that metals in the head of the Sharm sediments were of lithogenic origins, supplied to the Sharm either naturally by aeolian transport at one sampling site, with the exception of Pb. Some sampling sites in the mouth of the Sharm recorded non-lithogenic Pb and their occurrences were linked to waste disposal and fossil fuel combustion. 

Based on sediments collected from Sharm Obhur and Abu Madafi, Pan et al. [48] found that the metal pollution was not important and only the fish market site was moderately contaminated by Zn, Cu, and Pb. In Al-Kharrar Lagoon, Hariri and Abu-Zied [28] found the highest concentrations of metals were mainly due to the influx of silicyclastics from wadies into the middle and south-eastern parts of the lagoon, where metals were specifically associated with salinity, pH, and mud.

Youssef and El-Sorogy [50] found that the Pb and Cd concentrations surpassed the background values of the Al-Kharrar lagoon. In the northern and southern sections of the lagoon, elevated metal levels were found They also revealed that the Al-Kharrar lagoon’s bottom sediments were mildly polluted with Pb. Badr et al. [31] revealed that the most polluted region was Jeddah, followed by Rabigh, while the least contaminated area was Yanbu.

#### 4.9.3. Coasts of Yanbu and Aqaba

Based on mangrove sediments, Alzahrani et al. [75] found that the highest concentrations of the metals were above the levels of the threshold effects, suggesting a limited impact on the ecosystems concerned. Alharbi et al. [5] concluded that HMs polluted the marine sediments from the mangrove zone of the Red Sea at Yanbu, which might affect the quality of aquatic life and human beings.

#### 4.9.4. Aqaba Coasts

Based on thirty-three surface sediments collected from Aqaba Coast, El-Sorogy et al. [29] showed that Fe, Mn, Cd, Cu, Co, Zn, and Cr were predominantly of earthly origins, while As, Sb, Hg, Ni, and Pb were mainly due to traffic pollution, industrial activities, and dredging of marine sediments for anthropogenic activities. Al-Shami et al. [52] classified the Haqal coastal waters (Gulf of Aqaba) as ‘low ecological risk’. 

#### 4.9.5. Farasan Islands

Fawzy et al. [102] reported that, except for Cd, Pb, and Zn, the overall trace metal concentrations in the sediment samples were lower than in previous studies, induced by rising human activities on the Farasan Islands and landfilling operations [51]. Usman et al. [51] found that the maximum and mean concentrations of Cd, Cu, and Pb in the Farasan Island mangrove surface sediments exceeded their global average shale concentrations. In addition, only the highest concentration of Zn surpassed the global average concentration of shale. Based on the sediment quality guidelines (SQGs), the sediment samples collected were moderate to high for Cu, and non-polluted to heavy for Pb and Zn.

## 5. Conclusions

This paper calculated the values of PERI and HHRA of Cd, Cu, Pb, and Zn based on publications from the four Red Sea countries, with similar exposure factors and reference values. As compared to the reference values and UCC values, the average values of Cd, Cu, Pb, and Zn in the sediments were found to be higher. Out of the 68 reports, there were 32 reports (47.1%) exceeding PERI > 300 with ‘considerable ecological risk’ while there were 23 reports (33.8%) exceeding PERI > 600 with ‘very high ecological risk’. The HI values of Cd, Cu, Pb, and Zn for both children and adults were lower than 1 in all the reports, indicating limited non-carcinogenic risk from the four HMs in Red Sea countries. The NCR values (HI values < 1.0) of Cd, Cu, Pb, and Zn represented high no non-carcinogenic risks for the ingestion and dermal contact routes for sediments from the Red Sea countries. However, report no. 34 had the maximum Pb level (865 mg/kg) for the Hurghada region. It exceeded HI > 1.0, indicating Pb non-carcinogenic risk in this region. It can be concluded that the HM pollution in coastal surface sediments from the Red Sea countries were localized subjecting to highly anthropogenic sources in the vicinity or observable sources of human activities. Thus, the Sediment Watch program could be proposed [103]. This paper should be useful for researchers in evaluating environmental quality and the techniques discussed in it could be used for measuring and mitigating pollution in the Red Sea area holistically. 

## Figures and Tables

**Table 1:** Concentrations (mg/kg dry weight) of Cd, Cu, Pb, and Zn reported from countries in the Red Sea obtained from the literature.

Country	No.	Locations	Cu	Zn	Pb	Cd	References
Egypt	1	Northern Red Sea, 1984 (mean)	33.0	40.0	15.2	1.10	[2]
	2	North of Suez Gulf (Port Tawifiq, El-Kabanon, Nat. Inst. of Oceanogr. and Fish., Adabyia, and Ain Sukhna) (min)	1.84	4.26	13.9	2.26	[34]
	3	North of Suez Gulf (Port Tawifiq, El-Kabanon, Nat. Inst. of Oceanogr. and Fish., Adabyia, and Ain Sukhna) (max)	10.3	23.7	28.3	4.40	[34]
	4	Wadi Khashir (mean)	12.9	24.3	31.9	0.31	[35]
	5	Wadi El-Gemal (mean)	51.3	93.5	54.0	0.08	[35]
	6	Quseir Harbour (mean)	35.8	79.6	48.2	2.01	[36]
	7	Abutartour Harbour (mean)	46.7	91.7	63.3	2.50	[36]
	8	Touristic Harbour (mean)	21.3	47.7	39.0	2.97	[36]
	9	Hurghada Harbour (mean)	108	458	75.0	3.83	[36]
	10	Aqaba Gulf (Taba, Nuweiba, Dahab, Na’ama Bay, Ras Mohamed, and Sakalet Sharm) (min)	0.16	6.69	1.07	0.05	[37]
	11	Aqaba Gulf (Taba, Nuweiba, Dahab, Na’ama Bay, Ras Mohamed, and Sakalet Sharm) (max)	2.06	60.7	2.59	0.30	[37]
	12	Suez Gulf (El Tur, Ras Suder, Suez, Ain Sukhn, and Ras Gharb) (min)	0.11	4.69	0.31	0.07	[37]
	13	Suez Gulf (El Tur, Ras Suder, Suez, Ain Sukhn, and Ras Gharb) (max)	0.48	120	2.69	0.17	[37]
	14	Middle of Red Sea (NIOF (Hurghada), Safaga, Quseir, Marsa Alam, Shalatin Rahaba and Bir Shalatin) (min)	0.05	2.16	0.57	0.04	[37]
	15	Middle of Red Sea (NIOF (Hurghada), Safaga, Quseir, Marsa Alam, Shalatin Rahaba and Bir Shalatin) (max)	0.78	51.3	5.54	0.36	[37]
	16	Shalatein coastal area (min)	3.67	14.9	3.17	0.12	[38]
	17	Shalatein coastal area (max)	17.3	134	40.3	1.25	[38]
	18	Hurghada National Institute of Oceanography and Fisheries (30 sites)	1.26	7.77	42.4	0.14	[39]
	19	Egyptian Red Sea coast (mean)	1.94	22.6	3.26	0.10	[40]
	20	Hurghada area (El-Esh, Abu-Shaar, NIOF, Tourist Harbour, El-Samaka Village and Desert Rose Resort) (min)	0.05	0.01	0.01	0.03	[41]
	21	Hurghada area (El-Esh, Abu-Shaar, NIOF, Tourist Harbour, El-Samaka Village and Desert Rose Resort) (max)	23.3	49.4	9.83	0.68	[41]
	22	Hurghada City, Northern Red Sea (min)	10.5	21.0	30.0	2.50	[42]
	23	Hurghada City, Northern Red Sea (max)	78.0	150	53.0	4.00	[42]
	24	Ras Mohamed (mean)	9.45	20.8	11.2	1.07	[30]
	25	Um al-Sid (mean)	9.70	22.5	17.8	1.21	[30]
	26	Hurghada (mean)	56.0	110	54.51	1.66	[30]
	27	Qusier (mean)	18.5	31.3	31.8	10.20	[30]
	28	Gulf of Suez (Port Tawfiq, Rex Beach, Kabanon Beach, Suez Middle (NIOF), Ain Sukhna, Ain Sukhna, Ras Gharib, and Ras Shukeir, El-Tur) (mean)	7.71	85.3	47.8	3.26	[43]
	29	Gulf of Aqaba (Ras Mohamed, Marina Sharm, Sharm El Sheikh, Dahab, Nuweiba Harbor, and Taba) (mean)	8.86	58.2	31.2	2.43	[43]
	30	Red Sea proper (Hurghada, Safaga North, Safaga Middle, Hamarawein, Movenpick, Qusier Middle, Qusier South, Marsa Alam, and Bir Shalatin) (mean)	17.92	75.04	44.4	4.30	[43]
	31	Wadi El-Gemal area (min)	5.00	18.0	13.0	0.02	[44]
	32	Wadi El-Gemal area (max)	454	283	96.0	0.16	[44]
	33	Hurghada region (min)	0.20	10.7	38.0	0.07	[3]
	34	Hurghada region (max)	5.75	515	865	2.75	[3]
	35	Mabahiss Bay, North Hurghada (min)	5.30	2.80	10.7	0.50	[45]
	36	Mabahiss Bay, North Hurghada (max)	47.7	39.9	254	21.60	[45]
Saudi	1	Jeddah, Red Sea (min)	43.0	241	68.0	1.08	[26]
Arabia	2	Jeddah, Red Sea (max)	92.0	532	240	2.55	[26]
	3	Gulf of Aqaba (min)	7.60	7.00	3.70	0.06	[46]
	4	Gulf of Aqaba (max)	10.8	7.70	6.80	0.07	[46]
	5	Rabigh and Yanbu (min)	17.4	41.4	74.6	2.26	[31]
	6	Rabigh and Yanbu (max)	25.8	93.9	92.9	3.95	[31]
	7	Jeddah (min)	17.5	52.7	80.3	3.10	[31]
	8	Jeddah (max)	23.8	76.4	98.8	3.50	[31]
	9	Gulf of Aqaba (min)	23.0	17.0	3.50	0.50	[29]
	10	Gulf of Aqaba (max)	42.0	32.0	9.10	1.80	[29]
	11	Jazan coastal area (mean)	31.6	28.5	2.31	0.51	[47]
	12	Duba and Sharma, northwest Saudi Arabia (min)	5.60	7.70	1.50	0.10	[47]
	13	Duba and Sharma, northwest Saudi Arabia (max)	33.0	27.1	5.70	0.26	[47]
	14	Sharm Obhur to Abu Madafi Eastern coast of the Red Sea (min)	0.45	4.90	0.46	0.02	[48]
	15	Sharm Obhur to Abu Madafi Eastern coast of the Red Sea (max)	83.0	179	69.4	0.24	[48]
	16	Duba, Rabigh, Thuwal offshore, Jeddah, Farasan Banks/Al Qunfudhah, Jazan Economic Cit, Jazan and Farasan Islands (min)	0.17	3.82	1.23	0.10	[49]
	17	Duba, Rabigh, Thuwal offshore, Jeddah, Farasan Banks/Al Qunfudhah, Jazan Economic Cit, Jazan and Farasan Islands (max)	37.4	155	9.22	0.45	[49]
	18	Al-Kharrar lagoon, Rabigh, Saudi Arabia (mean)	16.0	39.7	50.9	0.26	[50]
	19	Mangrove area of Farasan Islands (mean)	111	57.2	45.2	1.23	[51]
	20	Al-Kharrar Lagoon (mean)	23.6	22.4	0.05	0.31	[28]
	21	Salman Bay (mean)	8.90	7.45	0.14	0.26	[28]
	22	Haqal coastal waters, Tabuk (mean)	1.13	4.52	2.64	0.19	[52]
Yemen	Y1	Al Hodeidah coast Yemen (12 sites) (mean)	61.0	11.5	5.61	3.04	[53]
	Y2	Background of Al-Hodeidah coast (mean)	51.1	5.10	5.80	2.50	[53]
	Y3	Yemen coast (Al-Luhayah, Al-Khawbah, Ibn Abbas village, Al-Salif, Ras Isa, Urj village, Ras Katib, Al-Mehwat, and Al-Taif beach) (min)	25.2	33.1	3.60	1.10	[54]
	Y4	Yemen coast (Al-Luhayah, Al-Khawbah, Ibn Abbas village, Al-Salif, Ras Isa, Urj village, Ras katib, Al-Mehwat, and Al-Taif beach) (max)	84.8	91.7	17.7	7.30	[54]
	Y5	Al-Khawakhah (mean)	3.60	8.50	3.50	0.70	[55]
	Y6	Al-Hodiedah (mean)	7.20	9.30	2.40	0.20	[55]
	Y7	Ras Katib (mean)	6.50	9.60	2.60	0.30	[55]
	Y8	Al-Urj (mean)	8.20	1.60	3.60	0.35	[55]
	Y9	Kamaran Island (mean)	7.90	7.60	4.60	0.40	[55]
Jordan	J1	Red Sea (North) Gulf of Aqaba (mean)	8.00	42.43	96.67	3.33	[56]
		Upper continental crust	14.3	52.0	17.0	0.10	[15]

Note: min= minimum; max= maximum.

**Table 2:** Definition, exposure factors, and reference values used to estimate the intake values and health risks of heavy metals in sediments for the present study.

Factor	Definition	Unit	Values		References
			Children	Adults	
IngR	Ingestion rate of soil	mg/day	200	100	[61]
BW	Body weight of the exposed individual	Kg	15	55.9	[63]
EF	Exposure frequency	days/year	350	350	[63]
ED	Exposure duration	Years	6	24	[61]
AT	Average time	Days	365ED	365ED	[59]
PEF	Particle emission factor	m^3^/kg	1.36 × 10^9^	1.36 × 10^9^	[61]
SA	Exposed skin surface area	cm^2^	1600	4350	[63]
AF	Skin adherence factor	mg/cm day	0.2	0.7	[64]
ABF	Dermal absorption factor	Unitless	0.001	0.001	[65]

**Table 3:** Descriptive statistics of heavy metal concentrations (mg/kg dry weight), concentration factors (Cf), ecological risks (Er) and potential ecological risk index (PERI) in the surface sediments of different countries in the Red Sea, in comparison to the established background values. (Numbers in brackets show the number of reports in each country).

Egypt (36)	Cu	Zn	Pb	Cd	Cf Cu	Cf Zn	Cf Pb	Cf Cd	Er Cu	Er Zn	Er Pb	Er Cd	PERI
Min	0.05	0.01	0.01	0.02	0.00	0.00	0.00	0.20	0.02	0.00	0.00	6.00	9.00
Max	454	515	865	21.6	31.8	9.9	50.9	216	159	9.90	254	6480	6572
Mean	30.8	77.2	57.8	2.18	2.15	1.49	3.40	21.8	10.8	1.49	17.0	654	683
Median	9.57	39.95	30.60	1.09	0.67	0.77	1.80	10.9	3.35	0.77	9.00	326	337
Std Error	12.8	19.2	24.2	0.65	0.89	0.37	1.42	6.50	4.46	0.37	7.12	195	197
Skewness	4.90	2.71	5.02	3.78	4.90	2.71	5.02	3.78	4.90	2.71	5.02	3.78	3.76
Kurtosis	24.5	6.92	25.08	15.7	24.5	6.92	25.1	15.7	24.5	6.92	25.1	15.7	15.6
SA (22)	Cu	Zn	Pb	Cd	Cf Cu	Cf Zn	Cf Pb	Cf Cd	Er Cu	Er Zn	Er Pb	Er Cd	PERI
Min	0.17	3.82	0.05	0.02	0.01	0.07	0.00	0.20	0.06	0.07	0.01	6.00	6.40
Max	111	532	240	3.95	7.80	10.2	14.1	39.5	38.8	10.2	70.6	1185	1223
Mean	29.8	74.5	39.7	1.04	2.08	1.43	2.32	10.4	10.4	1.43	11.6	310.91	334
Median	23.3	30.3	7.95	0.38	1.63	0.59	0.47	3.80	8.15	0.59	2.34	114	139
Std Error	6.37	25.6	12.2	0.27	0.45	0.49	0.71	2.65	2.23	0.49	3.57	79.6	82.9
Skewness	1.48	2.79	2.07	1.17	1.48	2.79	2.10	1.17	1.48	2.79	2.10	1.17	1.12
Kurtosis	1.37	7.78	4.71	−0.05	1.36	7.79	4.89	−0.05	1.37	7.79	4.88	−0.05	−0.20
Yemen (9)	Cu	Zn	Pb	Cd	Cf Cu	Cf Zn	Cf Pb	Cf Cd	Er Cu	Er Zn	Er Pb	Er Cd	PERI
Min	3.60	1.60	2.40	0.20	0.25	0.03	0.14	2.00	1.26	0.03	0.71	60.0	63.4
Max	84.8	91.7	17.7	7.30	5.93	1.76	1.04	73.0	29.7	1.76	5.21	2190	2227
Mean	28.4	19.8	5.49	1.77	1.98	0.38	0.32	17.7	9.93	0.38	1.62	530	542
Median	8.20	9.30	3.60	0.70	0.57	0.18	0.21	7.00	2.87	0.18	1.06	210	213
Std Error	9.96	9.47	1.58	0.77	0.70	0.18	0.09	7.71	3.48	0.18	0.46	231	235
Skewness	0.86	2.08	2.19	1.69	0.86	2.07	2.18	1.69	0.86	2.07	2.19	1.69	1.68
Kurtosis	−0.76	2.84	3.30	1.71	−0.76	2.82	3.29	1.71	−0.76	2.82	3.30	1.71	1.68
Jordan (1)	8.00	42.43	96.67	3.33	0.32	0.65	6.44	33.3	1.6	0.65	32.2	999	1033
Background values	Cu	Zn	Pb	Cd	Cf Cu	Cf Zn	Cf Pb	Cf Cd	Er Cu	Er Zn	Er Pb	Er Cd	PERI
Pre-industrial reference level ^1^	50.0	175	70.0	1.00	3.50	3.37	4.12	10.00	17.48	3.37	20.59	300.0	341.4
UCC ^2^	28.0	67.0	17.0	0.09	1.96	1.29	1.00	0.90	9.79	1.29	5.00	27.0	43.1
UCC ^3^	25.0	71.0	17.0	0.10	1.75	1.37	1.00	1.00	8.74	1.37	5.00	30.0	45.1
Background shale ^4^	45.0	95.0	20.0	0.30	3.15	1.83	1.18	3.00	15.73	1.83	5.88	90.0	113.4
UCC ^5^	25.0	65.0	15.0	0.10	1.75	1.25	0.88	1.00	8.74	1.25	4.41	30.0	44.4
UCC ^6^	14.3	52.0	17.0	0.10	1.00	1.00	1.00	1.00	5.00	1.00	5.00	30.0	41.0

Note: 1 = Hakanson [12]; 2 = Rudnick and Gao [17]; 3 = Taylor and McLennan [16]; 4 = Turekian and Wedepohl [14]; 5 = Wedepohl [70]; 6 = Wedepohl [15].

**Table 4:** Overall statistics of values of hazard quotient dermal (HQ_dermal_) and hazard quotient ingestion (HQ_ing_), and hazard index of Cd, Cu, Pb, and Zn for children and adults from the present study. N = 68.

Cd	Child HQ_ing_	Child HQ_dermal_	Child HI	Adult HQ_ing_	Adult HQ_dermal_	Adult HI
Minimum	2.62 × 10^−4^	4.19 × 10^−5^	3.04 × 10^−4^	3.52× 10^−5^	1.07 × 10^−4^	1.42 × 10^−4^
Maximum	3.79 × 10^−1^	6.06 × 10^−2^	4.39 × 10^−1^	3.80 × 10^−2^	1.16 × 10^−1^	1.54× 10^−1^
Mean	2.84 × 10^−2^	4.54 × 10^−3^	3.29 × 10^−2^	3.12 × 10^−3^	9.50 × 10^−3^	1.26 × 10^−2^
Median	8.78 × 10^−3^	1.41 × 10^−3^	1.02 × 10^−2^	1.05 × 10^−3^	3.19 × 10^−3^	4.24 × 10^−3^
Std Error	7.14 × 10^−3^	1.14 × 10^−3^	8.27 × 10^−3^	6.53 × 10^−4^	1.99 × 10^−3^	2.64 × 10^−3^
Skewness	4.47	4.47	4.46	4.47	4.48	4.47
Kurtosis	2.16 × 10	2.16 × 10	2.16 × 10	2.48 × 10	2.49 × 10	2.49 × 10
Pb	Child HQ_ing_	Child HQ_dermal_	Child HI	Adult HQ_ing_	Adult HQ_dermal_	Adult HI
Minimum	2.59 × 10^−5^	2.80 × 10^−7^	2.62 × 10^−5^	3.48 × 10^−6^	7.14 × 10^−7^	4.19 × 10^−6^
Maximum	3.20	3.46 × 10^−2^	3.24	4.30 × 10^−1^	8.82 × 10^−2^	5.18E × 10^−1^
Mean	1.69E × 10^−1^	1.82 × 10^−3^	1.71 × 10^−1^	2.26 × 10^−2^	4.65 × 10^−3^	2.73 × 10^−2^
Median	4.98 × 10^−2^	5.37 × 10^−4^	5.04 × 10^−2^	6.68 × 10^−3^	1.38 × 10^−3^	8.05 × 10^−3^
Std Error	5.00 × 10^−2^	5.40 × 10^−4^	5.06 × 10^−2^	6.71 × 10^−3^	1.38 × 10^−3^	8.09 × 10^−3^
Skewness	6.14	6.14	6.14	6.15	6.14	6.14
Kurtosis	4.16 × 10	4.17 × 10	4.17 × 10	4.17 × 10	4.17 × 10	4.17 × 10
Cu	Child HQ_ing_	Child HQ_dermal_	Child HI	Adult HQ_ing_	Adult HQ_dermal_	Adult HI
Minimum	1.64 × 10^−5^	8.74 × 10^−8^	1.65 × 10^−5^	2.20 × 10^−6^	2.23E × 10^−7^	2.42 × 10^−6^
Maximum	1.49 × 10^−1^	7.94 × 10^−4^	1.50 × 10^−1^	2.00 × 10^−2^	2.03 × 10^−3^	2.20 × 10^−2^
Mean	9.76 × 10^−3^	5.21 × 10^−5^	9.82 × 10^−3^	1.31 × 10^−3^	1.33 × 10^−4^	1.44 × 10^−3^
Median	3.89 × 10^−3^	2.07 × 10^−5^	3.91 × 10^−3^	5.21 × 10^−4^	5.29 × 10^−5^	5.74 × 10^−4^
Std Error	2.34 × 10^−3^	1.25 × 10^−5^	2.35 × 10^−3^	3.14 × 10^−4^	3.19 × 10^−5^	3.46 × 10^−4^
Skewness	5.74	5.74	5.75	5.74	5.74	5.74
Kurtosis	3.83 × 10	3.82× 10	3.83× 10	3.83× 10	3.83× 10	3.82× 10
Zn	Child HQ_ing_	Child HQ_dermal_	Child HI	Adult HQ_ing_	Adult HQ_dermal_	Adult HI
Minimum	4.37 × 10^−7^	3.50 × 10^−9^	4.40 × 10^−7^	5.86 × 10^−8^	8.93 × 10^−9^	6.76 × 10^−8^
Maximum	2.32 × 10^−2^	1.86 × 10^−4^	2.34 × 10^−2^	3.12 × 10^−3^	4.75 × 10^−4^	3.59 × 10^−3^
Mean	2.98 × 10^−3^	2.38 × 10^−5^	3.01 × 10^−3^	4.00 × 10^−4^	6.09 × 10^−5^	4.61 × 10^−4^
Median	1.31 × 10^−3^	1.04 × 10^−5^	1.32 × 10^−3^	1.75 × 10^−4^	2.67 × 10^−5^	2.02 × 10^−4^
Std Error	5.79 × 10^−4^	4.63 × 10^−6^	5.84 × 10^−4^	7.78 × 10^−5^	1.18 × 10^−5^	8.95 × 10^−5^
Skewness	2.98	2.99	2.98	2.99	2.99	2.98
Kurtosis	8.95	8.97	8.95	8.97	8.96	8.96

## Data Availability

Not applicable.

## Supplementary Materials

The following are available online at https://www.mdpi.com/1660-4601/18/6/2798/s1, Tables S1: Values of concentration factors (Cf), ecological risk (Er), and potential ecological risk index (PERI) calculated from the present study based on the cited concentrations of Cd, Cu, Pb and Zn reported from the Red Sea. Table S2. Values of hazard quotient ingestion (HQ_ing_), hazard quotient dermal (HQ_dermal_) and hazard index (HI) of Cd for children and adults from the present study. Table S3. Values of hazard quotient ingestion (HQ_ing_), hazard quotient dermal (HQ_dermal_) and hazard index (HI) of Cu for children and adults from the present study. Table S4. Values of hazard quotient ingestion (HQ_ing_), hazard quotient dermal (HQ_dermal_) and hazard index (HI) of Pb for children and adults from the present study. Table S5. Values of hazard quotient ingestion (HQ_ing_), hazard quotient dermal (HQ_dermal_) and hazard index (HI) of Zn for children and adults from the present study.
