# Supplementary material for: A Review of Heavy Metals in Coastal Surface Sediments from the Red Sea: Health-Ecological Risk Assessments
Source: Int J Environ Res Public Health. 2021 Mar 10;18(6):2798. doi: 10.3390/ijerph18062798 (PMC8000497; doi:10.3390/ijerph18062798)
Supplement: Supplementary file 1 [file ijerph-18-02798-s001.pdf]

Table S1. Values of concentration factors (Cf), ecological risk (Er), and potential ecological risk index (PERI) calculated from the present study based on the cited concentrations of Cd, Cu, Pb and Zn reported from the Red Sea.

Country	No.	Cf Cu	Cf Zn	Cf Pb	Cf Cd	Er Cu	Er Zn	Er Pb	Er Cd	PERI
Egypt	1	2.31	0.77	0.89	11.00	11.54	0.77	4.47	330.0	347
	2	0.13	0.08	0.82	22.60	0.64	0.08	4.09	678.0	683
	3	0.72	0.46	1.66	44.00	3.60	0.46	8.32	1320.0	1332
	4	0.90	0.47	1.88	3.10	4.51	0.47	9.38	93.0	107
	5	3.59	1.80	3.18	0.80	17.94	1.80	15.88	24.0	59.6
	6	2.50	1.53	2.84	20.10	12.52	1.53	14.18	603.0	631
	7	3.27	1.76	3.72	25.00	16.33	1.76	18.62	750.0	787
	8	1.49	0.92	2.29	29.70	7.45	0.92	11.47	891	911
	9	7.55	8.81	4.41	38.30	37.76	8.81	22.06	1149	1218
	10	0.01	0.13	0.06	0.50	0.06	0.13	0.31	15.0	15.5
	11	0.14	1.17	0.15	3.00	0.72	1.17	0.76	90.0	92.6
	12	0.01	0.09	0.02	0.70	0.04	0.09	0.09	21.0	21.2
	13	0.03	2.31	0.16	1.70	0.17	2.31	0.79	51.0	54.3
	14	0.00	0.04	0.03	0.40	0.02	0.04	0.17	12.0	12.2
	15	0.05	0.99	0.33	3.60	0.27	0.99	1.63	108.0	110.9
	16	0.26	0.29	0.19	1.20	1.28	0.29	0.93	36.0	38.5
	17	1.21	2.58	2.37	12.50	6.05	2.58	11.85	375.0	396
	18	0.09	0.15	2.49	1.40	0.44	0.15	12.47	42.0	55.1
	19	0.14	0.43	0.19	1.00	0.68	0.43	0.96	30.0	32.1
	20	0.00	0.00	0.00	0.30	0.02	0.00	0.00	9.0	9.0
	21	1.63	0.95	0.58	6.80	8.15	0.95	2.89	204.0	216
	22	0.73	0.40	1.76	25.00	3.67	0.40	8.82	750.0	763
	23	5.45	2.88	3.12	40.00	27.27	2.88	15.59	1200.0	1246
	24	0.66	0.40	0.66	10.70	3.30	0.40	3.29	321.0	328
	25	0.68	0.43	1.05	12.10	3.39	0.43	5.24	363.0	372
	26	3.92	2.12	3.21	16.60	19.58	2.12	16.03	498.0	536
	27	1.29	0.60	1.87	102.00	6.47	0.60	9.35	3060.0	3076
	28	0.54	1.64	2.81	32.60	2.70	1.64	14.06	978.0	996
	29	0.62	1.12	1.84	24.30	3.10	1.12	9.18	729.0	742
	30	1.25	1.44	2.61	43.00	6.27	1.44	13.06	1290.0	1311
	31	0.35	0.35	0.76	0.20	1.75	0.35	3.82	6.0	11.9
	32	31.75	5.44	5.65	1.60	158.74	5.44	28.24	48.0	240
	33	0.01	0.21	2.24	0.70	0.07	0.21	11.18	21.0	32.5
	34	0.40	9.90	50.88	27.50	2.01	9.90	254.41	825.0	1091
	35	0.37	0.05	0.63	5.00	1.85	0.05	3.15	150.0	155
	36	3.34	0.77	14.94	216.00	16.68	0.77	74.71	6480.0	6572
Saudi Arabia	1	3.01	4.63	4.00	10.80	15.03	4.63	20.00	324.0	364
	2	6.43	10.23	14.12	25.50	32.17	10.23	70.59	765.0	878
	3	0.53	0.13	0.22	0.60	2.66	0.13	1.09	18.0	21.9
	4	0.76	0.15	0.40	0.70	3.78	0.15	2.00	21.0	26.9
	5	1.22	0.80	4.39	22.60	6.08	0.80	21.94	678.0	707
	6	1.80	1.81	5.46	39.50	9.02	1.81	27.32	1185.0	1223
	7	1.22	1.01	4.72	31.00	6.12	1.01	23.62	930.0	961
	8	1.66	1.47	5.81	35.00	8.32	1.47	29.06	1050.0	1089
	9	1.61	0.33	0.21	5.00	8.04	0.33	1.03	150.0	159
	10	2.94	0.62	0.54	18.00	14.69	0.62	2.68	540.0	558
	11	2.21	0.55	0.14	5.10	11.05	0.55	0.68	153.0	165
	12	0.39	0.15	0.09	1.00	1.96	0.15	0.44	30.0	32.5
	13	2.31	0.52	0.34	2.60	11.54	0.52	1.68	78.0	91.7
	14	0.03	0.09	0.03	0.20	0.16	0.09	0.14	6.0	6.40
	15	5.80	3.44	4.08	2.40	29.02	3.44	20.41	72.0	125
	16	0.01	0.07	0.07	1.00	0.06	0.07	0.36	30.0	30.5
	17	2.62	2.98	0.54	4.50	13.08	2.98	2.71	135.0	154
	18	1.12	0.76	2.99	2.60	5.59	0.76	14.97	78.0	99.3
	19	7.76	1.10	2.66	12.30	38.81	1.10	13.29	369.0	422
	20	1.65	0.43	0.00	3.10	8.25	0.43	0.01	93.0	102
	21	0.62	0.14	0.01	2.60	3.11	0.14	0.04	78.0	81.3
	22	0.08	0.09	0.16	1.90	0.40	0.09	0.78	57.0	58.3
Yemen	Y1	4.27	0.22	0.33	30.40	21.33	0.22	1.65	912.0	935

	Y2	3.57	0.10	0.34	25.00	17.87	0.10	1.71	750.0	770
	Y3	1.76	0.64	0.21	11.00	8.81	0.64	1.06	330.0	341
	Y4	5.93	1.76	1.04	73.00	29.65	1.76	5.21	2190.0	2227
	Y5	0.25	0.16	0.21	7.00	1.26	0.16	1.03	210.0	213
	Y6	0.50	0.18	0.14	2.00	2.52	0.18	0.71	60.0	63.4
	Y7	0.45	0.18	0.15	3.00	2.27	0.18	0.76	90.0	93.2
	Y8	0.57	0.03	0.21	3.50	2.87	0.03	1.06	105.0	109
	Y9	0.55	0.15	0.27	4.00	2.76	0.15	1.35	120.0	124
Jordan	J1	0.56	0.82	5.69	33.30	2.80	0.82	28.43	999.0	1031
UCC		1.00	1.00	1.00	1.00	5.00	1.00	5.00	30.0	41.0

Note: SA= Saudi Arabia; UCC= Upper continental crust background levels by Wedepohl [1].

Table S2. Values of hazard quotient ingestion (HQ_{ing}), hazard quotient dermal (HQ_{dermal}) and hazard index (HI) of Cd for children and adults from the present study. N= 68.

		**Children**			**Adults**		
Country	No.	HQ_{ing}	HQ_{dermal}	HI	HQ_{ing}	HQ_{dermal}	HI
Egypt	1	2.96E-02	4.74E-03	3.44E-02	1.93E-03	5.89E-03	7.83E-03
	2	5.77E-02	9.23E-03	6.69E-02	3.97E-03	1.21E-02	1.61E-02
	3	3.79E-01	6.06E-02	4.39E-01	7.74E-03	2.36E-02	3.13E-02
	4	1.05E-03	1.68E-04	1.22E-03	5.45E-04	1.66E-03	2.21E-03
	5	2.63E-02	4.22E-03	3.06E-02	1.41E-04	4.28E-04	5.69E-04
	6	3.28E-02	5.24E-03	3.80E-02	3.54E-03	1.08E-02	1.43E-02
	7	3.89E-02	6.23E-03	4.52E-02	4.40E-03	1.34E-02	1.78E-02
	8	5.02E-02	8.03E-03	5.82E-02	5.22E-03	1.59E-02	2.11E-02
	9	6.55E-04	1.05E-04	7.60E-04	6.74E-03	2.05E-02	2.72E-02
	10	3.93E-03	6.29E-04	4.56E-03	8.79E-05	2.68E-04	3.56E-04
	11	9.18E-04	1.47E-04	1.06E-03	5.28E-04	1.61E-03	2.13E-03
	12	2.23E-03	3.57E-04	2.59E-03	1.23E-04	3.75E-04	4.98E-04
	13	5.24E-04	8.39E-05	6.08E-04	2.99E-04	9.10E-04	1.21E-03
	14	4.72E-03	7.55E-04	5.47E-03	7.04E-05	2.14E-04	2.85E-04
	15	1.57E-03	2.52E-04	1.82E-03	6.33E-04	1.93E-03	2.56E-03
	16	1.64E-02	2.62E-03	1.90E-02	2.11E-04	6.43E-04	8.54E-04
	17	1.84E-03	2.94E-04	2.13E-03	2.20E-03	6.69E-03	8.89E-03
	18	1.34E-03	2.14E-04	1.55E-03	2.46E-04	7.50E-04	9.96E-04
	19	1.34E-03	2.14E-04	1.55E-03	1.79E-04	5.46E-04	7.26E-04
	20	8.91E-03	1.43E-03	1.03E-02	5.28E-05	1.61E-04	2.13E-04
	21	3.28E-02	5.24E-03	3.80E-02	1.20E-03	3.64E-03	4.84E-03
	22	5.24E-02	8.39E-03	6.08E-02	4.40E-03	1.34E-02	1.78E-02
	23	1.40E-02	2.24E-03	1.63E-02	7.04E-03	2.14E-02	2.85E-02
	24	1.59E-02	2.54E-03	1.84E-02	1.88E-03	5.73E-03	7.61E-03
	25	2.18E-02	3.48E-03	2.52E-02	2.13E-03	6.48E-03	8.61E-03
	26	1.34E-01	2.14E-02	1.55E-01	2.92E-03	8.89E-03	1.18E-02
	27	4.27E-02	6.84E-03	4.96E-02	1.79E-02	5.46E-02	7.26E-02
	28	3.19E-02	5.10E-03	3.70E-02	5.73E-03	1.75E-02	2.32E-02
	29	5.63E-02	9.01E-03	6.53E-02	4.28E-03	1.30E-02	1.73E-02
	30	2.62E-04	4.19E-05	3.04E-04	7.56E-03	2.30E-02	3.06E-02
	31	2.10E-03	3.36E-04	2.43E-03	3.52E-05	1.07E-04	1.42E-04
	32	9.18E-04	1.47E-04	1.06E-03	2.81E-04	8.57E-04	1.14E-03
	33	3.60E-02	5.77E-03	4.18E-02	1.23E-04	3.75E-04	4.98E-04
	34	6.55E-03	1.05E-03	7.60E-03	4.84E-03	1.47E-02	1.96E-02
	35	2.83E-01	4.53E-02	3.28E-01	8.79E-04	2.68E-03	3.56E-03
	36	3.01E-03	4.82E-04	3.50E-03	3.80E-02	1.16E-01	1.54E-01
Saudi Arabia	1	3.34E-02	5.35E-03	3.88E-02	1.90E-03	5.78E-03	7.68E-03
	2	7.87E-04	1.26E-04	9.12E-04	4.48E-03	1.37E-02	1.81E-02
	3	9.18E-04	1.47E-04	1.06E-03	1.06E-04	3.21E-04	4.27E-04
	4	2.96E-02	4.74E-03	3.44E-02	1.23E-04	3.75E-04	4.98E-04
	5	5.18E-02	8.28E-03	6.01E-02	3.97E-03	1.21E-02	1.61E-02
	6	4.06E-02	6.50E-03	4.71E-02	6.95E-03	2.12E-02	2.81E-02
	7	4.59E-02	7.34E-03	5.32E-02	5.45E-03	1.66E-02	2.21E-02
	8	6.55E-03	1.05E-03	7.60E-03	6.16E-03	1.87E-02	2.49E-02
	9	2.36E-02	3.78E-03	2.74E-02	8.79E-04	2.68E-03	3.56E-03
	10	9.31E-03	1.49E-03	1.08E-02	3.17E-03	9.64E-03	1.28E-02
	11	1.31E-03	2.10E-04	1.52E-03	8.97E-04	2.73E-03	3.63E-03

	12	3.41E-03	5.45E-04	3.95E-03	1.76E-04	5.36E-04	7.11E-04
	13	2.62E-04	4.19E-05	3.04E-04	4.57E-04	1.39E-03	1.85E-03
	14	3.15E-03	5.03E-04	3.65E-03	3.52E-05	1.07E-04	1.42E-04
	15	1.31E-03	2.10E-04	1.52E-03	4.22E-04	1.29E-03	1.71E-03
	16	5.90E-03	9.44E-04	6.84E-03	1.76E-04	5.36E-04	7.11E-04
	17	3.41E-03	5.45E-04	3.95E-03	7.91E-04	2.41E-03	3.20E-03
	18	8.65E-03	1.38E-03	1.00E-02	4.57E-04	1.39E-03	1.85E-03
	19	4.06E-03	6.50E-04	4.71E-03	2.16E-03	6.59E-03	8.75E-03
	20	3.41E-03	5.45E-04	3.95E-03	5.45E-04	1.66E-03	2.21E-03
	21	3.99E-02	6.38E-03	4.62E-02	4.57E-04	1.39E-03	1.85E-03
	22	3.28E-02	5.24E-03	3.80E-02	3.34E-04	1.02E-03	1.35E-03
Yemen	Y1	3.28E-02	5.24E-03	3.80E-02	5.35E-03	1.63E-02	2.16E-02
	Y2	1.44E-02	2.31E-03	1.67E-02	4.40E-03	1.34E-02	1.78E-02
	Y3	9.57E-02	1.53E-02	1.11E-01	1.93E-03	5.89E-03	7.83E-03
	Y4	9.18E-03	1.47E-03	1.06E-02	1.28E-02	3.91E-02	5.19E-02
	Y5	2.62E-03	4.19E-04	3.04E-03	1.23E-03	3.75E-03	4.98E-03
	Y6	3.93E-03	6.29E-04	4.56E-03	3.52E-04	1.07E-03	1.42E-03
	Y7	4.59E-03	7.34E-04	5.32E-03	5.28E-04	1.61E-03	2.13E-03
	Y8	5.24E-03	8.39E-04	6.08E-03	6.16E-04	1.87E-03	2.49E-03
	Y9	5.24E-03	8.39E-04	6.08E-03	7.04E-04	2.14E-03	2.85E-03
Jordan	J1	1.44E-02	2.31E-03	1.67E-02	5.86E-03	1.78E-02	2.37E-02
UCC		2.96E-02	4.74E-03	3.44E-02	1.76E-04	5.36E-04	7.11E-04

Note: SA= Saudi Arabia; UCC= Upper continental crust background levels by Wedepohl [1].

Table S3. Values of hazard quotient ingestion (HQ_{ing}), hazard quotient dermal (HQ_{dermal}) and hazard index (HI) of Cu for children and adults from the present study.

		**Children**			**Adults**		
Country	No.	HQ_{ing}	HQ_{dermal}	HI	HQ_{ing}	HQ_{dermal}	HI
Egypt	1	1.08E-02	5.77E-05	1.09E-02	1.45E-03	1.47E-04	1.60E-03
	2	6.03E-04	3.22E-06	6.06E-04	8.09E-05	8.21E-06	8.91E-05
	3	3.36E-03	1.79E-05	3.38E-03	4.51E-04	4.57E-05	4.96E-04
	4	4.23E-03	2.25E-05	4.25E-03	5.67E-04	5.76E-05	6.25E-04
	5	1.68E-02	8.97E-05	1.69E-02	2.26E-03	2.29E-04	2.48E-03
	6	1.17E-02	6.26E-05	1.18E-02	1.57E-03	1.60E-04	1.73E-03
	7	1.53E-02	8.16E-05	1.54E-02	2.05E-03	2.08E-04	2.26E-03
	8	6.98E-03	3.72E-05	7.02E-03	9.37E-04	9.51E-05	1.03E-03
	9	3.54E-02	1.89E-04	3.56E-02	4.75E-03	4.82E-04	5.23E-03
	10	5.24E-05	2.80E-07	5.27E-05	7.04E-06	7.14E-07	7.75E-06
	11	6.75E-04	3.60E-06	6.79E-04	9.06E-05	9.19E-06	9.98E-05
	12	3.60E-05	1.92E-07	3.62E-05	4.84E-06	4.91E-07	5.33E-06
	13	1.57E-04	8.39E-07	1.58E-04	2.11E-05	2.14E-06	2.32E-05
	14	1.64E-05	8.74E-08	1.65E-05	2.20E-06	2.23E-07	2.42E-06
	15	2.56E-04	1.36E-06	2.57E-04	3.43E-05	3.48E-06	3.78E-05
	16	1.20E-03	6.41E-06	1.21E-03	1.61E-04	1.64E-05	1.78E-04
	17	5.68E-03	3.03E-05	5.71E-03	7.62E-04	7.73E-05	8.39E-04
	18	4.13E-04	2.20E-06	4.15E-04	5.54E-05	5.62E-06	6.10E-05
	19	6.36E-04	3.39E-06	6.39E-04	8.53E-05	8.66E-06	9.40E-05
	20	1.64E-05	8.74E-08	1.65E-05	2.20E-06	2.23E-07	2.42E-06
	21	7.62E-03	4.06E-05	7.66E-03	1.02E-03	1.04E-04	1.13E-03
	22	3.44E-03	1.84E-05	3.46E-03	4.62E-04	4.69E-05	5.09E-04
	23	2.56E-02	1.36E-04	2.57E-02	3.43E-03	3.48E-04	3.78E-03
	24	3.10E-03	1.65E-05	3.11E-03	4.16E-04	4.22E-05	4.58E-04
	25	3.18E-03	1.70E-05	3.20E-03	4.26E-04	4.33E-05	4.70E-04
	26	1.84E-02	9.79E-05	1.84E-02	2.46E-03	2.50E-04	2.71E-03
	27	6.08E-03	3.24E-05	6.11E-03	8.15E-04	8.27E-05	8.98E-04
	28	2.53E-03	1.35E-05	2.54E-03	3.39E-04	3.44E-05	3.73E-04
	29	2.90E-03	1.55E-05	2.92E-03	3.90E-04	3.96E-05	4.29E-04
	30	5.87E-03	3.13E-05	5.90E-03	7.88E-04	8.00E-05	8.68E-04
	31	1.64E-03	8.74E-06	1.65E-03	2.20E-04	2.23E-05	2.42E-04
	32	1.49E-01	7.94E-04	1.50E-01	2.00E-02	2.03E-03	2.20E-02
	33	6.55E-05	3.50E-07	6.59E-05	8.79E-06	8.93E-07	9.69E-06
	34	1.88E-03	1.00E-05	1.89E-03	2.53E-04	2.57E-05	2.78E-04
	35	1.74E-03	9.26E-06	1.75E-03	2.33E-04	2.37E-05	2.57E-04

	36	1.56E-02	8.34E-05	1.57E-02	2.10E-03	2.13E-04	2.31E-03
Saudi Arabia	1	1.41E-02	7.52E-05	1.42E-02	1.89E-03	1.92E-04	2.08E-03
	2	3.01E-02	1.61E-04	3.03E-02	4.05E-03	4.11E-04	4.46E-03
	3	2.49E-03	1.33E-05	2.50E-03	3.34E-04	3.39E-05	3.68E-04
	4	3.54E-03	1.89E-05	3.56E-03	4.75E-04	4.82E-05	5.23E-04
	5	5.70E-03	3.04E-05	5.73E-03	7.65E-04	7.77E-05	8.43E-04
	6	8.46E-03	4.51E-05	8.50E-03	1.13E-03	1.15E-04	1.25E-03
	7	5.74E-03	3.06E-05	5.77E-03	7.69E-04	7.81E-05	8.48E-04
	8	7.79E-03	4.15E-05	7.83E-03	1.05E-03	1.06E-04	1.15E-03
	9	7.54E-03	4.02E-05	7.58E-03	1.01E-03	1.03E-04	1.11E-03
	10	1.38E-02	7.34E-05	1.38E-02	1.85E-03	1.87E-04	2.03E-03
	11	1.04E-02	5.52E-05	1.04E-02	1.39E-03	1.41E-04	1.53E-03
	12	1.84E-03	9.79E-06	1.84E-03	2.46E-04	2.50E-05	2.71E-04
	13	1.08E-02	5.77E-05	1.09E-02	1.45E-03	1.47E-04	1.60E-03
	14	1.47E-04	7.87E-07	1.48E-04	1.98E-05	2.01E-06	2.18E-05
	15	2.72E-02	1.45E-04	2.73E-02	3.65E-03	3.70E-04	4.02E-03
	16	5.57E-05	2.97E-07	5.60E-05	7.47E-06	7.59E-07	8.23E-06
	17	1.23E-02	6.54E-05	1.23E-02	1.64E-03	1.67E-04	1.81E-03
	18	5.24E-03	2.80E-05	5.27E-03	7.04E-04	7.14E-05	7.75E-04
	19	3.64E-02	1.94E-04	3.66E-02	4.88E-03	4.95E-04	5.38E-03
	20	7.74E-03	4.13E-05	7.78E-03	1.04E-03	1.05E-04	1.14E-03
	21	2.92E-03	1.56E-05	2.93E-03	3.91E-04	3.97E-05	4.31E-04
	22	3.70E-04	1.98E-06	3.72E-04	4.97E-05	5.04E-06	5.47E-05
Yemen	Y1	2.00E-02	1.07E-04	2.01E-02	2.68E-03	2.72E-04	2.96E-03
	Y2	1.67E-02	8.93E-05	1.68E-02	2.25E-03	2.28E-04	2.47E-03
	Y3	8.26E-03	4.40E-05	8.30E-03	1.11E-03	1.12E-04	1.22E-03
	Y4	2.78E-02	1.48E-04	2.79E-02	3.73E-03	3.78E-04	4.11E-03
	Y5	1.18E-03	6.29E-06	1.19E-03	1.58E-04	1.61E-05	1.74E-04
	Y6	2.36E-03	1.26E-05	2.37E-03	3.17E-04	3.21E-05	3.49E-04
	Y7	2.13E-03	1.14E-05	2.14E-03	2.86E-04	2.90E-05	3.15E-04
	Y8	2.69E-03	1.43E-05	2.70E-03	3.61E-04	3.66E-05	3.97E-04
	Y9	2.59E-03	1.38E-05	2.60E-03	3.47E-04	3.53E-05	3.83E-04
Jordan	J1	2.62E-03	1.40E-05	2.64E-03	3.52E-04	3.57E-05	3.87E-04
UCC		8.19E-03	4.37E-05	8.24E-03	1.10E-03	1.12E-04	1.21E-03

Note: SA= Saudi Arabia; UCC= Upper continental crust background levels by Wedepohl [1].

Table S4. Values of hazard quotient ingestion (HQ_{ing}), hazard quotient dermal (HQ_{dermal}) and hazard index (HI) of Pb for children and adults from the present study.

		Children			Adults		
Country	No.	HQ_{ing}	HQ_{dermal}	HI	HQ_{ing}	HQ_{dermal}	HI
Egypt	1	5.63E-02	6.07E-04	5.69E-02	7.55E-03	1.55E-03	9.10E-03
	2	5.15E-02	5.55E-04	5.20E-02	6.91E-03	1.42E-03	8.32E-03
	3	1.05E-01	1.13E-03	1.06E-01	1.41E-02	2.89E-03	1.70E-02
	4	1.18E-01	1.28E-03	1.19E-01	1.59E-02	3.26E-03	1.91E-02
	5	2.00E-01	2.16E-03	2.02E-01	2.68E-02	5.51E-03	3.23E-02
	6	1.78E-01	1.93E-03	1.80E-01	2.39E-02	4.92E-03	2.89E-02
	7	2.34E-01	2.53E-03	2.37E-01	3.14E-02	6.46E-03	3.79E-02
	8	1.44E-01	1.56E-03	1.46E-01	1.94E-02	3.98E-03	2.34E-02
	9	2.78E-01	3.00E-03	2.81E-01	3.73E-02	7.65E-03	4.49E-02
	10	3.96E-03	4.27E-05	4.00E-03	5.32E-04	1.09E-04	6.41E-04
	11	9.59E-03	1.03E-04	9.69E-03	1.29E-03	2.64E-04	1.55E-03
	12	1.15E-03	1.24E-05	1.16E-03	1.54E-04	3.16E-05	1.86E-04
	13	9.96E-03	1.07E-04	1.01E-02	1.34E-03	2.74E-04	1.61E-03
	14	2.11E-03	2.28E-05	2.13E-03	2.83E-04	5.81E-05	3.41E-04
	15	2.05E-02	2.21E-04	2.07E-02	2.75E-03	5.65E-04	3.32E-03
	16	1.17E-02	1.27E-04	1.19E-02	1.57E-03	3.23E-04	1.90E-03
	17	1.49E-01	1.61E-03	1.51E-01	2.00E-02	4.11E-03	2.41E-02
	18	1.57E-01	1.69E-03	1.59E-01	2.11E-02	4.32E-03	2.54E-02
	19	1.21E-02	1.30E-04	1.22E-02	1.62E-03	3.33E-04	1.95E-03
	20	2.59E-05	2.80E-07	2.62E-05	3.48E-06	7.14E-07	4.19E-06
	21	3.64E-02	3.93E-04	3.68E-02	4.88E-03	1.00E-03	5.89E-03
	22	1.11E-01	1.20E-03	1.12E-01	1.49E-02	3.06E-03	1.80E-02

	23	1.96E-01	2.12E-03	1.98E-01	2.63E-02	5.41E-03	3.17E-02
	24	4.16E-02	4.49E-04	4.21E-02	5.58E-03	1.15E-03	6.73E-03
	25	6.59E-02	7.11E-04	6.66E-02	8.84E-03	1.82E-03	1.07E-02
	26	2.02E-01	2.18E-03	2.04E-01	2.71E-02	5.56E-03	3.26E-02
	27	1.18E-01	1.27E-03	1.19E-01	1.58E-02	3.24E-03	1.90E-02
	28	1.77E-01	1.91E-03	1.79E-01	2.37E-02	4.87E-03	2.86E-02
	29	1.16E-01	1.25E-03	1.17E-01	1.55E-02	3.19E-03	1.87E-02
	30	1.64E-01	1.77E-03	1.66E-01	2.20E-02	4.53E-03	2.66E-02
	31	4.81E-02	5.19E-04	4.87E-02	6.46E-03	1.33E-03	7.78E-03
	32	3.55E-01	3.84E-03	3.59E-01	4.77E-02	9.79E-03	5.75E-02
	33	1.41E-01	1.52E-03	1.42E-01	1.89E-02	3.88E-03	2.28E-02
	34	3.20E+00	3.46E-02	3.24E+00	4.30E-01	8.82E-02	5.18E-01
	35	3.96E-02	4.27E-04	4.00E-02	5.32E-03	1.09E-03	6.41E-03
	36	9.42E-01	1.02E-02	9.52E-01	1.26E-01	2.59E-02	1.52E-01
Saudi Arabia	1	2.52E-01	2.72E-03	2.55E-01	3.38E-02	6.94E-03	4.07E-02
	2	8.89E-01	9.59E-03	8.98E-01	1.19E-01	2.45E-02	1.44E-01
	3	1.37E-02	1.48E-04	1.38E-02	1.84E-03	3.77E-04	2.22E-03
	4	2.52E-02	2.72E-04	2.55E-02	3.38E-03	6.94E-04	4.07E-03
	5	2.76E-01	2.98E-03	2.79E-01	3.71E-02	7.61E-03	4.47E-02
	6	3.66E-01	3.95E-03	3.70E-01	4.91E-02	1.01E-02	5.92E-02
	7	2.97E-01	3.21E-03	3.01E-01	3.99E-02	8.19E-03	4.81E-02
	8	3.66E-01	3.95E-03	3.70E-01	4.91E-02	1.01E-02	5.92E-02
	9	1.30E-02	1.40E-04	1.31E-02	1.74E-03	3.57E-04	2.10E-03
	10	3.37E-02	3.64E-04	3.41E-02	4.52E-03	9.28E-04	5.45E-03
	11	8.55E-03	9.23E-05	8.65E-03	1.15E-03	2.36E-04	1.38E-03
	12	5.55E-03	5.99E-05	5.61E-03	7.45E-04	1.53E-04	8.98E-04
	13	2.11E-02	2.28E-04	2.13E-02	2.83E-03	5.81E-04	3.41E-03
	14	1.70E-03	1.84E-05	1.72E-03	2.29E-04	4.69E-05	2.75E-04
	15	2.57E-01	2.77E-03	2.60E-01	3.45E-02	7.08E-03	4.15E-02
	16	4.55E-03	4.91E-05	4.60E-03	6.11E-04	1.25E-04	7.37E-04
	17	3.41E-02	3.68E-04	3.45E-02	4.58E-03	9.41E-04	5.52E-03
	18	1.88E-01	2.03E-03	1.90E-01	2.53E-02	5.19E-03	3.05E-02
	19	1.67E-01	1.81E-03	1.69E-01	2.25E-02	4.61E-03	2.71E-02
	20	1.85E-04	2.00E-06	1.87E-04	2.48E-05	5.10E-06	2.99E-05
	21	5.18E-04	5.59E-06	5.24E-04	6.96E-05	1.43E-05	8.38E-05
	22	9.78E-03	1.05E-04	9.88E-03	1.31E-03	2.69E-04	1.58E-03
Yemen	Y1	2.08E-02	2.24E-04	2.10E-02	2.79E-03	5.72E-04	3.36E-03
	Y2	2.15E-02	2.32E-04	2.17E-02	2.88E-03	5.92E-04	3.47E-03
	Y3	1.33E-02	1.44E-04	1.35E-02	1.79E-03	3.67E-04	2.16E-03
	Y4	6.55E-02	7.07E-04	6.63E-02	8.79E-03	1.81E-03	1.06E-02
	Y5	1.30E-02	1.40E-04	1.31E-02	1.74E-03	3.57E-04	2.10E-03
	Y6	8.89E-03	9.59E-05	8.98E-03	1.19E-03	2.45E-04	1.44E-03
	Y7	9.63E-03	1.04E-04	9.73E-03	1.29E-03	2.65E-04	1.56E-03
	Y8	1.33E-02	1.44E-04	1.35E-02	1.79E-03	3.67E-04	2.16E-03
	Y9	1.70E-02	1.84E-04	1.72E-02	2.29E-03	4.69E-04	2.75E-03
Jordan	J1	3.58E-01	3.86E-03	3.62E-01	4.80E-02	9.86E-03	5.79E-02
UCC		5.55E-02	5.99E-04	5.61E-02	7.45E-03	1.53E-03	8.98E-03

Note: SA= Saudi Arabia; UCC= Upper continental crust background levels by Wedepohl [1].

Table S5. Values of hazard quotient ingestion (HQ_{ing}), hazard quotient dermal (HQ_{dermal}) and hazard index (HI) of Zn for children and adults from the present study.

		**Children**			**Adults**		
Country	No.	HQ_{ing}	HQ_{dermal}	HI	HQ_{ing}	HQ_{dermal}	HI
Egypt	1	1.75E-03	1.40E-05	1.76E-03	2.35E-04	3.57E-05	2.70E-04
	2	1.86E-04	1.49E-06	1.88E-04	2.50E-05	3.80E-06	2.88E-05
	3	1.03E-03	8.28E-06	1.04E-03	1.39E-04	2.11E-05	1.60E-04
	4	1.06E-03	8.50E-06	1.07E-03	1.43E-04	2.17E-05	1.64E-04
	5	4.09E-03	3.27E-05	4.12E-03	5.48E-04	8.35E-05	6.32E-04
	6	3.48E-03	2.78E-05	3.51E-03	4.67E-04	7.10E-05	5.38E-04
	7	4.01E-03	3.21E-05	4.04E-03	5.38E-04	8.18E-05	6.19E-04
	8	2.08E-03	1.67E-05	2.10E-03	2.80E-04	4.26E-05	3.22E-04
	9	2.00E-02	1.60E-04	2.02E-02	2.69E-03	4.09E-04	3.09E-03

	10	2.92E-04	2.34E-06	2.95E-04	3.92E-05	5.97E-06	4.52E-05
	11	2.65E-03	2.12E-05	2.67E-03	3.56E-04	5.42E-05	4.10E-04
	12	2.05E-04	1.64E-06	2.07E-04	2.75E-05	4.19E-06	3.17E-05
	13	5.24E-03	4.19E-05	5.28E-03	7.03E-04	1.07E-04	8.10E-04
	14	9.44E-05	7.55E-07	9.51E-05	1.27E-05	1.93E-06	1.46E-05
	15	2.24E-03	1.79E-05	2.26E-03	3.01E-04	4.58E-05	3.47E-04
	16	6.53E-04	5.22E-06	6.58E-04	8.76E-05	1.33E-05	1.01E-04
	17	5.86E-03	4.69E-05	5.91E-03	7.87E-04	1.20E-04	9.07E-04
	18	3.40E-04	2.72E-06	3.42E-04	4.56E-05	6.94E-06	5.25E-05
	19	9.89E-04	7.91E-06	9.97E-04	1.33E-04	2.02E-05	1.53E-04
	20	4.37E-07	3.50E-09	4.40E-07	5.86E-08	8.93E-09	6.76E-08
	21	2.16E-03	1.73E-05	2.18E-03	2.90E-04	4.41E-05	3.34E-04
	22	9.18E-04	7.34E-06	9.25E-04	1.23E-04	1.87E-05	1.42E-04
	23	6.55E-03	5.24E-05	6.61E-03	8.79E-04	1.34E-04	1.01E-03
	24	9.11E-04	7.28E-06	9.18E-04	1.22E-04	1.86E-05	1.41E-04
	25	9.83E-04	7.87E-06	9.91E-04	1.32E-04	2.01E-05	1.52E-04
	26	4.79E-03	3.83E-05	4.82E-03	6.42E-04	9.78E-05	7.40E-04
	27	1.37E-03	1.09E-05	1.38E-03	1.83E-04	2.79E-05	2.11E-04
	28	3.73E-03	2.98E-05	3.76E-03	5.00E-04	7.61E-05	5.76E-04
	29	2.54E-03	2.04E-05	2.56E-03	3.41E-04	5.20E-05	3.93E-04
	30	3.28E-03	2.62E-05	3.31E-03	4.40E-04	6.70E-05	5.07E-04
	31	7.87E-04	6.29E-06	7.93E-04	1.06E-04	1.61E-05	1.22E-04
	32	1.24E-02	9.89E-05	1.25E-02	1.66E-03	2.53E-04	1.91E-03
	33	4.65E-04	3.72E-06	4.69E-04	6.24E-05	9.51E-06	7.19E-05
	34	2.25E-02	1.80E-04	2.27E-02	3.02E-03	4.60E-04	3.48E-03
	35	1.22E-04	9.79E-07	1.23E-04	1.64E-05	2.50E-06	1.89E-05
	36	1.74E-03	1.39E-05	1.76E-03	2.34E-04	3.56E-05	2.70E-04
Saudi Arabia	1	1.05E-02	8.42E-05	1.06E-02	1.41E-03	2.15E-04	1.63E-03
	2	2.32E-02	1.86E-04	2.34E-02	3.12E-03	4.75E-04	3.59E-03
	3	3.06E-04	2.45E-06	3.08E-04	4.10E-05	6.25E-06	4.73E-05
	4	3.36E-04	2.69E-06	3.39E-04	4.51E-05	6.87E-06	5.20E-05
	5	1.81E-03	1.45E-05	1.82E-03	2.43E-04	3.70E-05	2.80E-04
	6	4.10E-03	3.28E-05	4.14E-03	5.50E-04	8.38E-05	6.34E-04
	7	2.30E-03	1.84E-05	2.32E-03	3.09E-04	4.71E-05	3.56E-04
	8	3.34E-03	2.67E-05	3.36E-03	4.48E-04	6.82E-05	5.16E-04
	9	7.43E-04	5.94E-06	7.49E-04	9.97E-05	1.52E-05	1.15E-04
	10	1.40E-03	1.12E-05	1.41E-03	1.88E-04	2.86E-05	2.16E-04
	11	1.25E-03	9.97E-06	1.26E-03	1.67E-04	2.54E-05	1.93E-04
	12	3.36E-04	2.69E-06	3.39E-04	4.51E-05	6.87E-06	5.20E-05
	13	1.18E-03	9.47E-06	1.19E-03	1.59E-04	2.42E-05	1.83E-04
	14	2.14E-04	1.71E-06	2.16E-04	2.87E-05	4.37E-06	3.31E-05
	15	7.82E-03	6.26E-05	7.88E-03	1.05E-03	1.60E-04	1.21E-03
	16	1.67E-04	1.34E-06	1.68E-04	2.24E-05	3.41E-06	2.58E-05
	17	6.79E-03	5.43E-05	6.84E-03	9.11E-04	1.39E-04	1.05E-03
	18	1.74E-03	1.39E-05	1.75E-03	2.33E-04	3.54E-05	2.68E-04
	19	2.50E-03	2.00E-05	2.52E-03	3.35E-04	5.11E-05	3.86E-04
	20	9.80E-04	7.84E-06	9.87E-04	1.31E-04	2.00E-05	1.51E-04
	21	3.26E-04	2.60E-06	3.28E-04	4.37E-05	6.65E-06	5.03E-05
	22	1.98E-04	1.58E-06	1.99E-04	2.65E-05	4.03E-06	3.05E-05
Yemen	Y1	5.02E-04	4.02E-06	5.06E-04	6.74E-05	1.03E-05	7.76E-05
	Y2	2.23E-04	1.78E-06	2.25E-04	2.99E-05	4.55E-06	3.45E-05
	Y3	1.45E-03	1.16E-05	1.46E-03	1.94E-04	2.95E-05	2.24E-04
	Y4	4.01E-03	3.21E-05	4.04E-03	5.38E-04	8.18E-05	6.19E-04
	Y5	3.71E-04	2.97E-06	3.74E-04	4.98E-05	7.59E-06	5.74E-05
	Y6	4.06E-04	3.25E-06	4.10E-04	5.45E-05	8.30E-06	6.28E-05
	Y7	4.19E-04	3.36E-06	4.23E-04	5.63E-05	8.57E-06	6.48E-05
	Y8	6.99E-05	5.59E-07	7.05E-05	9.38E-06	1.43E-06	1.08E-05
	Y9	3.32E-04	2.66E-06	3.35E-04	4.46E-05	6.78E-06	5.13E-05
Jordan	J1	1.85E-03	1.48E-05	1.87E-03	2.49E-04	3.79E-05	2.87E-04
UCC		2.84E-03	2.27E-05	2.86E-03	3.81E-04	5.80E-05	4.39E-04

Note: SA= Saudi Arabia; UCC= Upper continental crust background levels by Wedepohl [1].

Reference

1. Wedepohl, K.H. The Composition of the Continental Crust. *Geochim. Cosmochim. Acta* **1995**, *59*, 1217–1232, doi:10.1016/0016-7037(95)00038-2.
